# Supplementary material for: S100A1 is released from ischemic cardiomyocytes and signals myocardial damage via Toll-like receptor 4
Source: EMBO Mol Med. 2014 May 15;6(6):778–94. doi: 10.15252/emmm.201303498 (PMC4203355; doi:10.15252/emmm.201303498)
Supplement: Supplementary file 1 — Supplementary Figure S1 [file emmm0006-0778-sd1.pdf]

Supplemental Figure I, Rohde et al.

A

	**controls**	**STEMI**
n	12	12
Age [yrs]	60±17	64±13
Male gender [%]	58	75
S100A1 [ng/mL]	3.0 (2.8;4.3)	48.4 (30.3;181.7)
hsTnT [ng/L]	13.0 (4.7;23.1)	4590.0 (683.5;9774.5)
CK [U/L]	95.5 (55.5;125.5)	766.0 (366.0;2449.5)
LDH [U/L]	171.5 (148.5;223.0)	484.0 (352.5;945.5)
CAD [%]	75	100
PCI performed	0	12
ASS	8	12
Dual platelet inhibition	1	12
Gp IIb/IIIa inhibitor	0	4
Beta blocker	7	9
ACE inh./AT1 antag.	8	6

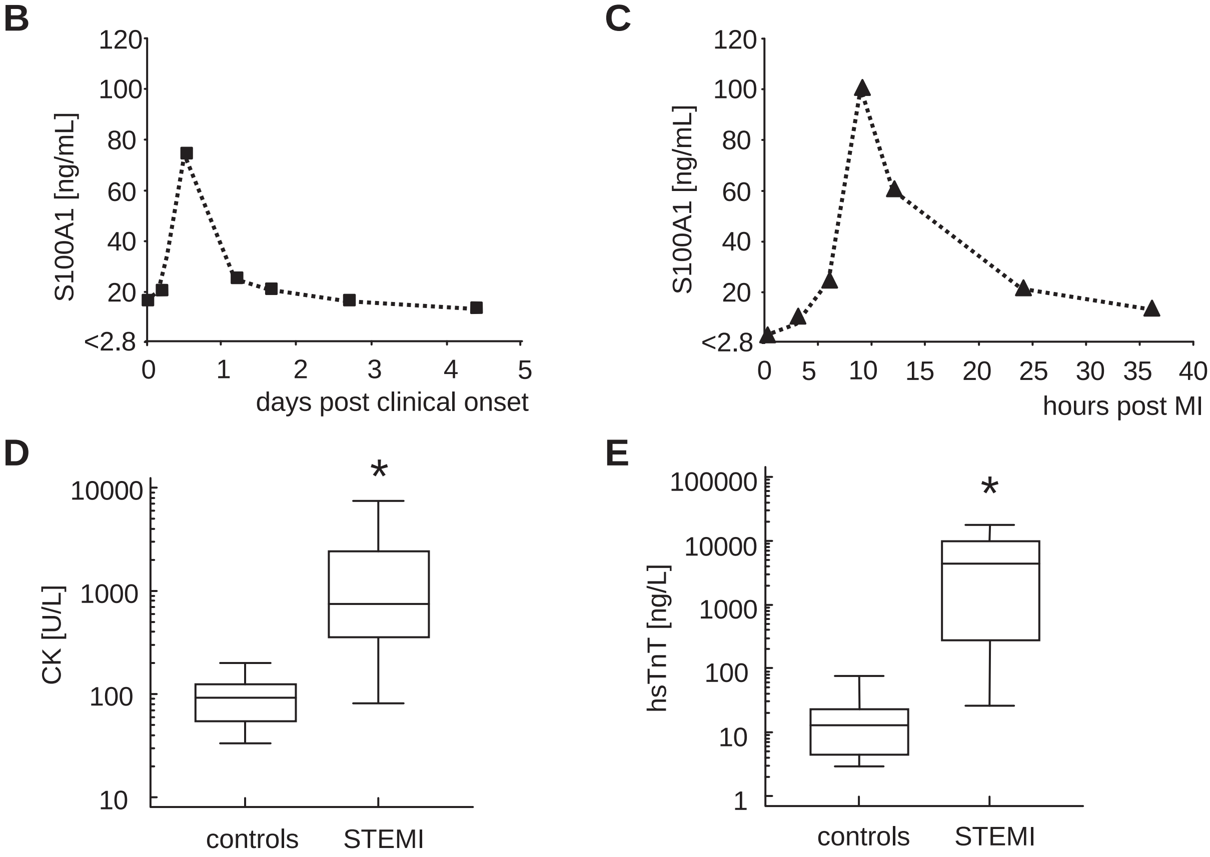

Supplemental Figure I. Patients' characteristics, S100A1 serum course and serum levels of creatin kinase and high-sensitive troponin T. A, Table with characteristics of patients presenting to the internal medicine emergency department without acute myocardial infarction (controls) and with ST-segment elevation myocardial infarction (STEMI) (LDH: lactate dehydrogenase; CAD: coronary artery disease; PCI: percutaneous coronary intervention; data displayed as median with 25th and 75th percentiles). **B**, Representative transient S100A1 serum course derived from one patient sampled by ELISA. **C**, Transient S100A1 serum course of one mouse. **D-E**, Serum levels of creatin kinase (CK) (**D**) and high-sensitive troponin T (hsTnT) (**E**) in the control and STEMI group confirmed necrosis of cardiomyocytes in STEMI patients (n=12 patients in each group, *P=0.02 for CK, *P=0.001 for hsTnT).
