# Supplementary material for: S100A1 is released from ischemic cardiomyocytes and signals myocardial damage via Toll-like receptor 4
Source: EMBO Mol Med. 2014 May 15;6(6):778–94. doi: 10.15252/emmm.201303498 (PMC4203355; doi:10.15252/emmm.201303498)
Supplement: Supplementary file 2 — Supplementary Figure S2 [file emmm0006-0778-sd2.pdf]

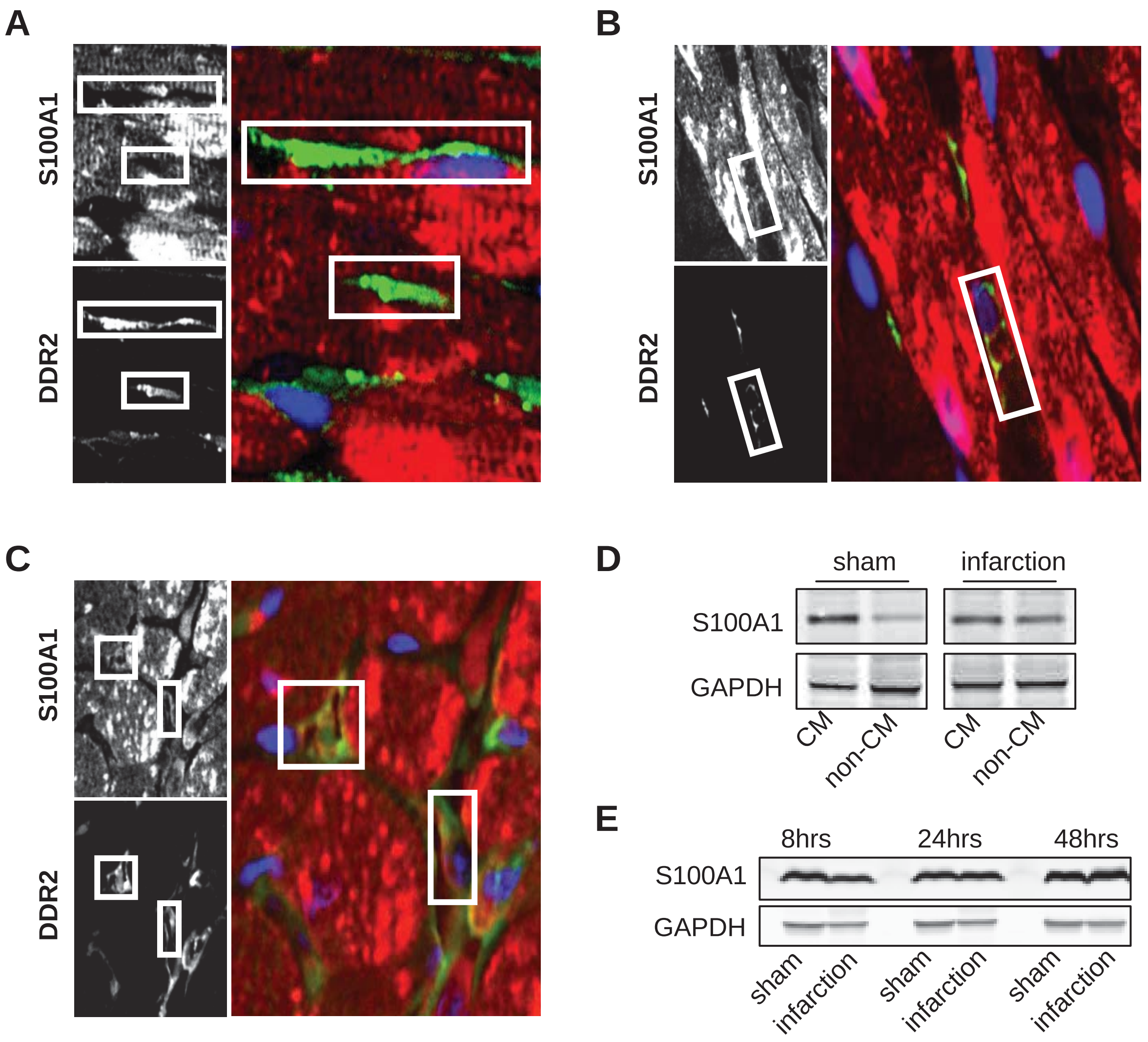

Supplemental Figure II. Internalization of S100A1 from necrotic cardiomyocytes by interstitial fibroblasts *in vivo*, S100A1 accumulation in the non-myocyte fraction and S100A1 content of remote myocardium post infarction. A, Representative immunofluorescence staining of sham-operated murine myocardium. S100A1 displays a striated pattern in cardiomyocytes (upper greyscale image and red in coloured merge). S100A1-negative interstitial cardiac fibroblasts are labelled with anti-discoidin domain receptorin 2 (DDR2) (lower greyscale image and green in coloured merge). DAPI/nuclei: blue. **B-C**, Representative IF stainings of infarcted murine myocardium (infarct border zone). In contrast to sham-operated myocardium, S100A1 is detectable in DDR2-labelled cardiac fibroblasts. **D**, Representative Western blot of cardiomyocyte (CM) and non-cardiomyocyte (non-CM) fractions from sham-operated (left) and infarcted (right) murine myocardium. As a result of myocardial infarction, S100A1 protein content is elevated in the non-CM fraction. **E**, Representative Western blot of samples taken from the left-ventricular posterior wall (non-infarcted, remote myocardium) of sham-operated and infarcted mice at different time points. No difference in S100A1 content was observed between the groups.
