# Supplementary material for: S100A1 is released from ischemic cardiomyocytes and signals myocardial damage via Toll-like receptor 4
Source: EMBO Mol Med. 2014 May 15;6(6):778–94. doi: 10.15252/emmm.201303498 (PMC4203355; doi:10.15252/emmm.201303498)
Supplement: Supplementary file 3 — Supplementary Figure S3 [file emmm0006-0778-sd3.pdf]

Supplemental Figure III, Rohde et al.

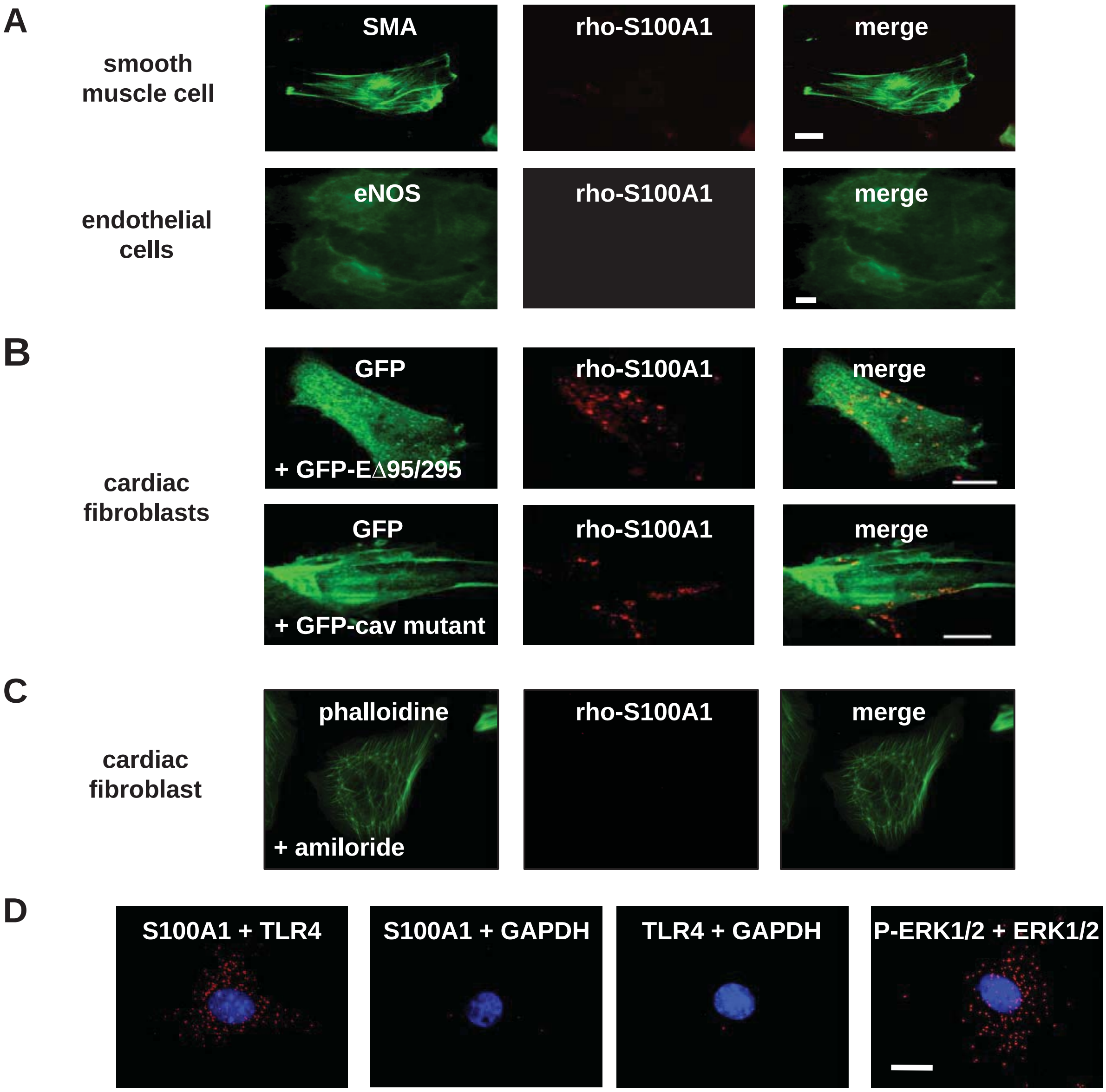

Supplemental Figure III. Exclusive S100A1 clathrin- and caveolin-independent internalization by fibroblasts and interaction with intracellular Toll-like receptor 4. A, Smooth muscle and endothelial cells show no uptake of rhodamine-labelled S100A1 (rho-S100A1: red). Counterstaining with smooth muscle actin (SMA: green) and endothelial NO synthase (eNOS: green). **B**, Cardiac fibroblasts with defective clathrin endocytosis (upper panel) or caveolin endocytosis (lower panel) still internalize rho-S100A1 (red). Cells were transfected with dominant-negative mutants for clathrin and caveolin, respectively, GFP (green) confirmed mutant expression. **C**, S100A1 uptake by cardiac fibroblasts was abolished in the presence of macropinocytosis-inhibitor amiloride. **D**, Representative proximity ligation assay images (Duolink®) showing molecular binding of S100A1 to intracellular Toll-like receptor 4 (TLR4) (far left). Red dots represent spots of protein-protein interaction (DAPI: blue). S100A1+GAPDH and TLR4+GAPDH served as negative controls (no signal). Usage of phospho-ERK1/2 and ERK1/2 antibodies directed against different epitopes of the same protein served as positive control (far right).
