# Supplementary material for: S100A1 is released from ischemic cardiomyocytes and signals myocardial damage via Toll-like receptor 4
Source: EMBO Mol Med. 2014 May 15;6(6):778–94. doi: 10.15252/emmm.201303498 (PMC4203355; doi:10.15252/emmm.201303498)
Supplement: Supplementary file 4 — Supplementary Figure S4 [file emmm0006-0778-sd4.pdf]

Supplemental Figure IV, Rohde et al.

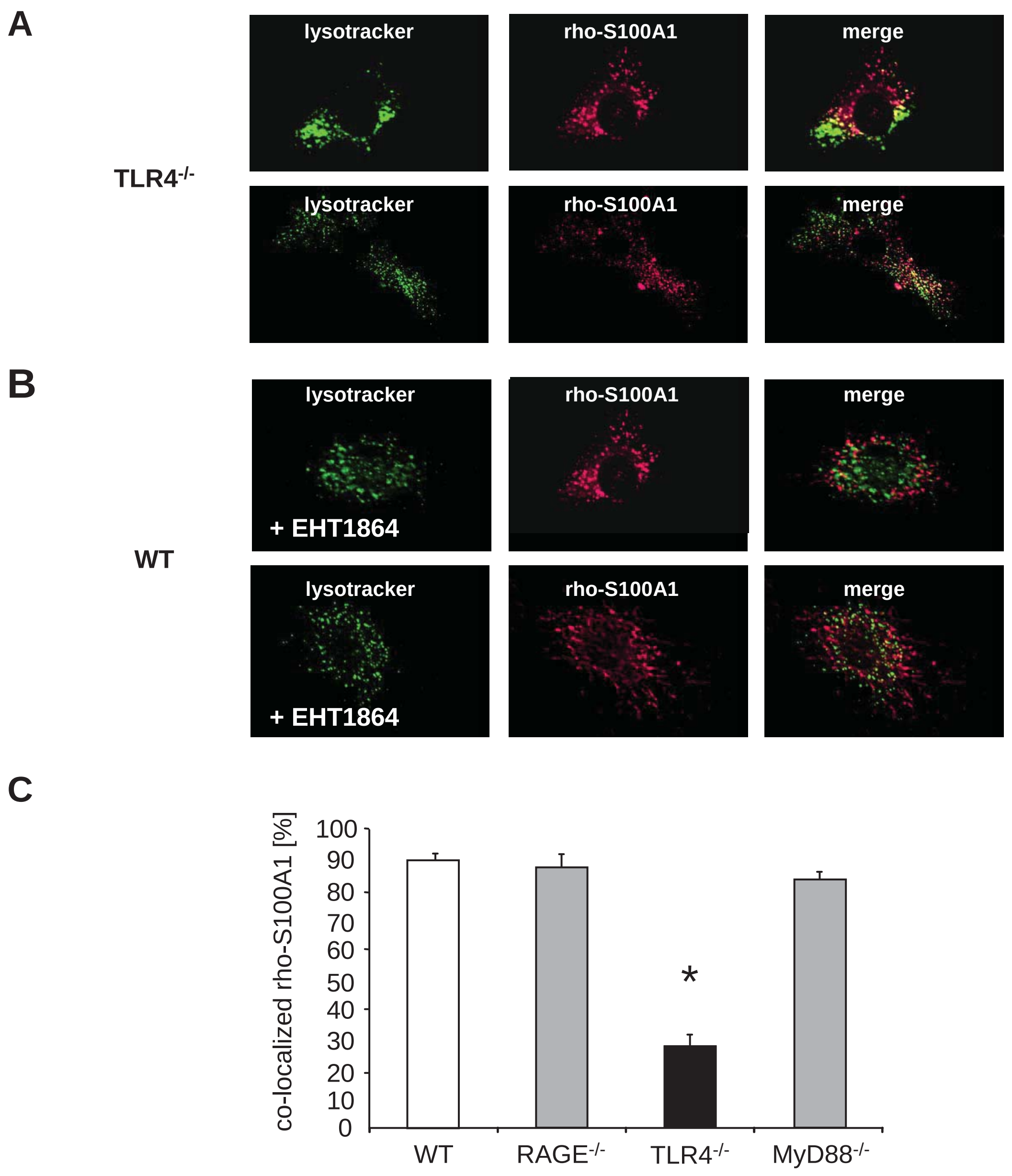

Supplemental Figure IV. TLR4- and Rac1-dependency of S100A1 endolysosomal trafficking. **A**, Additional images from TLR4-/- fibroblasts; no co-localization of S100A1 with lysotracker can be observed. **B**, Pre-treatment of WT cells with Rac1-inhibitor EHT1864 mimicks the results obtained in TLR4-/- cells. **C**, Statistical analysis of rho-S100A1 and FITC-lysotracker co-localization. Data are expressed as percentage of red rho-S100A1-dots that co-localize with green lysotracker in the merged images and therefore turn yellow. Analysis was performed using a standardized protocol of ImageJ-Software (*P=0.001, n=20 individual cells analysed in each group).
