# Supplementary material for: S100A1 is released from ischemic cardiomyocytes and signals myocardial damage via Toll-like receptor 4
Source: EMBO Mol Med. 2014 May 15;6(6):778–94. doi: 10.15252/emmm.201303498 (PMC4203355; doi:10.15252/emmm.201303498)
Supplement: Supplementary file 5 — Supplementary Figure S5 [file emmm0006-0778-sd5.pdf]

Supplemental Figure V, Rohde et al.

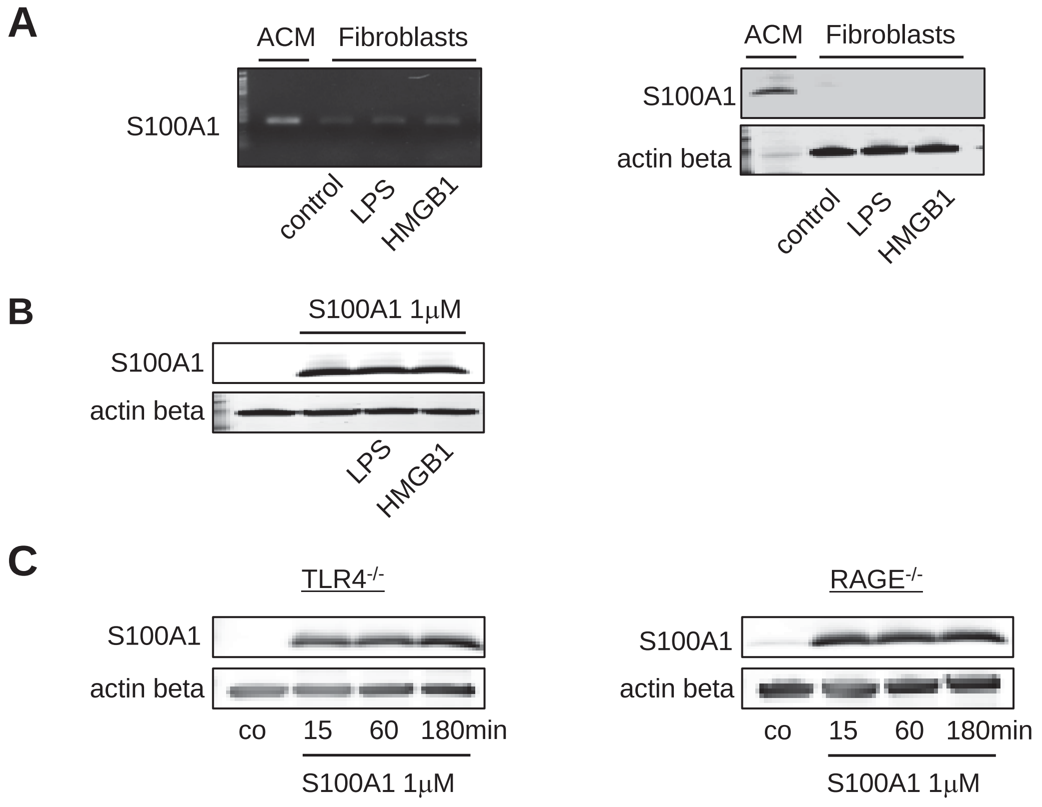

Supplemental Figure V. Expression of S100A1 and time-course of S100A1 internalization in TLR4-/- and RAGE-/- fibroblasts. A, Cardiac fibroblasts were analysed for S100A1 expression under basal conditions and following pre-treatment with LPS and HMGB1 for 24hrs (left panel: DNA gel from RT-PCR samples after 35 cycles of amplification, right panel: representative Western blot). Samples from adult cardiomyocytes (ACM) served as positive control. **B**, Pre-treatment of fibroblasts with LPS and HMGB1 for 24hrs does not alter the amount of internalized S100A1 (representative Western blot, fibroblasts were exposed to S100A1 for 30min). **C**, The time-course of S100A1 internalization in TLR4-/- and RAGE-/- fibroblasts shows no difference to WT cells, strengthening the notion of S100A1 internalization by receptor-independent fluid endocytosis (representative Western blots shown).
