# Supplementary material for: S100A1 is released from ischemic cardiomyocytes and signals myocardial damage via Toll-like receptor 4
Source: EMBO Mol Med. 2014 May 15;6(6):778–94. doi: 10.15252/emmm.201303498 (PMC4203355; doi:10.15252/emmm.201303498)
Supplement: Supplementary file 6 — Supplementary Figure S6 [file emmm0006-0778-sd6.pdf]

Supplemental Figure VI, Rohde et al.

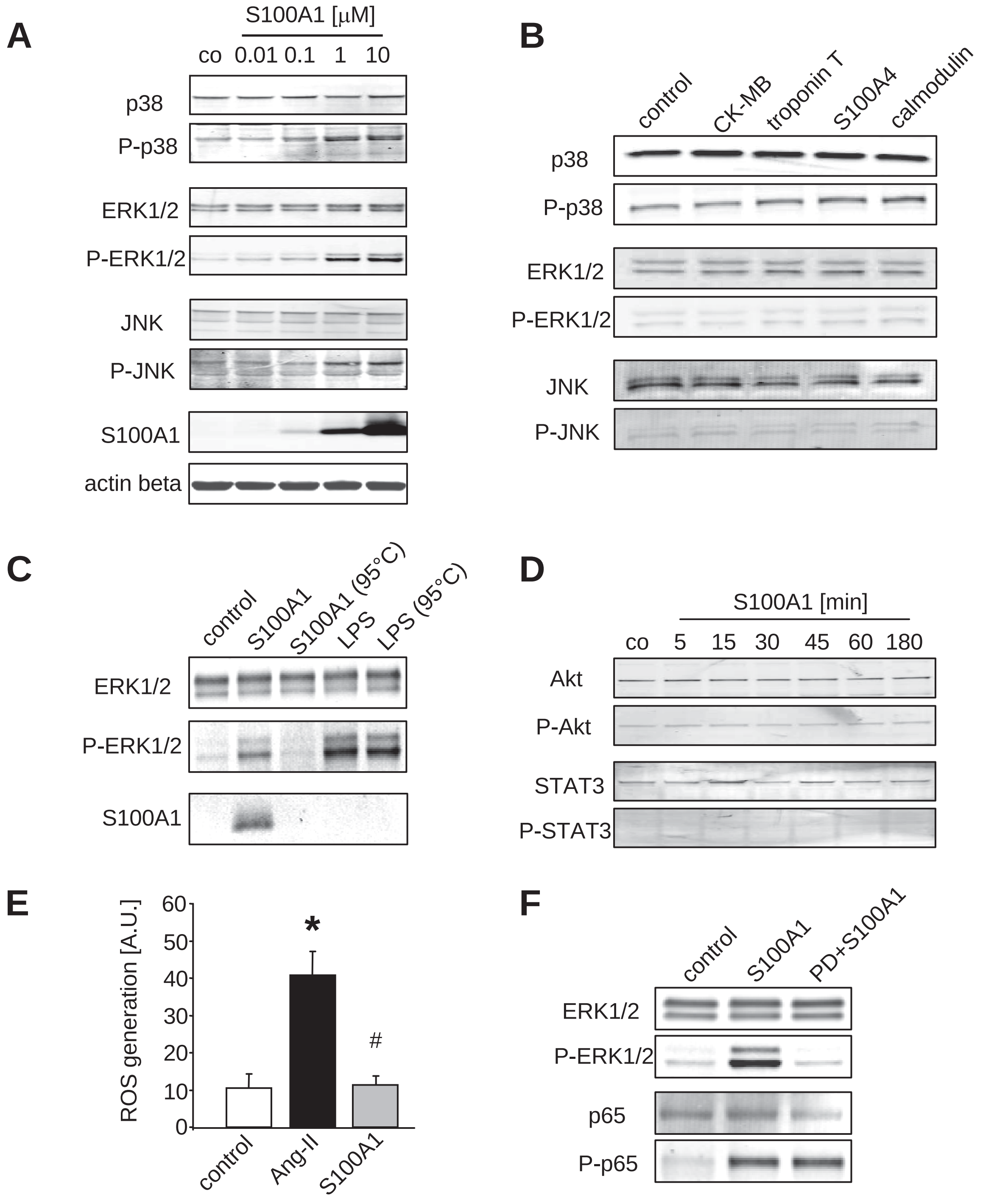

Supplemental Figure VI. Specific targeting of MAP-kinase and NFkappaB signalling pathways by S100A1 in cardiac fibroblasts. A, S100A1 mediates activation of mitogen- and stress-activated protein kinases (MAPK, SAPK) in a dose-dependent manner. Half maximal effective S100A1 concentrations (EC50) were below 1μM. **B**, Incubation of cardiac fibroblasts (CFs) with myocardial-type creatin kinase (CK-MB), troponin T, S100A4 and calmodulin (all 1μM for 30min) has no effect on MAPK/SAPK activation. **C**, Heating to 95°C for 10min leads to inactivation of S100A1 due to protein degradation. In contrast to S100A1, bacterial LPS still activates ERK1/2 in CFs, indicating no relevant contamination of recombinant S100A1 with LPS. **D**, Neither Akt nor STAT3 are phosphorylated in response to S100A1 in CFs. **E**, Incubation with S100A1 does not increase reactive oxygen species (ROS) generation in CFs. Angiotensin II (Ang-II) served as positive control (n=3 individual experiments, *P=0.01 vs control, #P=0.03 vs Ang-II). **F**, Pre-incubation with ERK1/2-inhibitor PD98059 (PD) has no influence on NFkappaB activation (displayed as p65 phosphorylation) in CFs, pointing towards MAPK-independent NFkappaB activation by S100A1.
