# Supplementary material for: S100A1 is released from ischemic cardiomyocytes and signals myocardial damage via Toll-like receptor 4
Source: EMBO Mol Med. 2014 May 15;6(6):778–94. doi: 10.15252/emmm.201303498 (PMC4203355; doi:10.15252/emmm.201303498)
Supplement: Supplementary file 7 — Supplementary Figure S7 [file emmm0006-0778-sd7.pdf]

Supplemental Figure VII, Rohde et al.

A

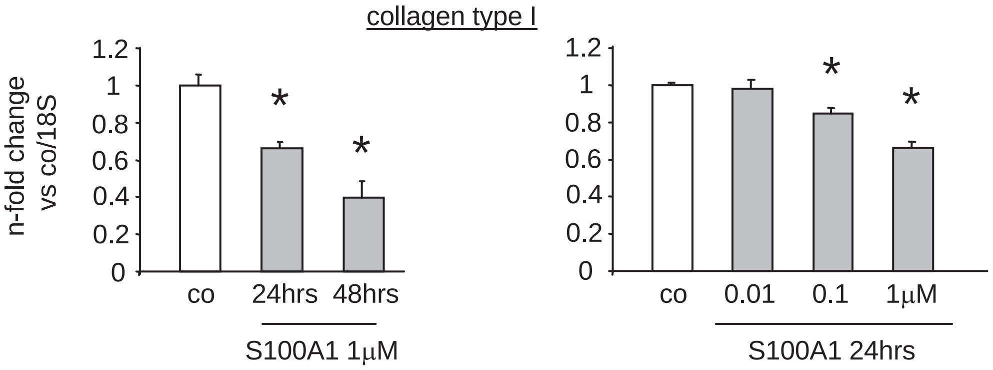

B

	n-fold change vs control	SEM	P vs control
**chemokines**			
monocyte chemotactic protein 1	71.24	0.33	0.0052
macrophage inflammatory protein 1a	41.00	1.82	0.0024
macrophage migration inhibitory factor	3.89	0.26	0.0001
TGF-b1	0.27	0.06	0.0140
**interleukins**			
interleukin 1 alpha	636.74	22.40	0.0084
interleukin 11	205.60	16.52	0.0036
**extracellular matrix proteins**			
collagen type III	0.12	0.09	0.0255
matrix metalloproteinase 2	2.59	0.12	0.0061
tissue inhibitor of metalloproteinase 3	0.38	0.27	0.0130
fibronectin 1	1.17	0.25	0.6593
**adhesion molecules**			
vascular cell adhesion protein 1	5.29	1.56	0.0002
platelet endothelial cell adhesion molecule 1	1.95	0.30	0.0312
cadherin 1	3.29	0.94	0.1302

C D

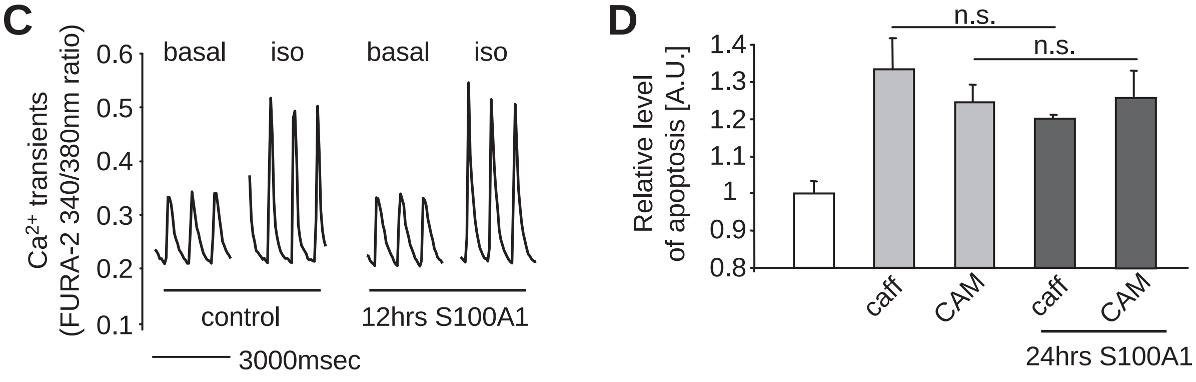

Supplemental Figure VII. Extracellular S100A1 time- and dose-dependent cardiac fibroblast gene expression and S100A1 effect on cardiomyocyte Ca^{2+} homeostasis and susceptibility to apoptosis. A, Collagen type I gene expression is diminished in response to S100A1 in a time- (left panel) and dose- (right panel) dependent manner (left: *P=values vs co: 0.04 and 0.02; right *P-values vs co: 0.04 and 0.03; n=5). **B**, Extracellular S100A1 diversely affects gene expression in cardiac fibroblasts (presented as n-fold change in mRNA level, SEM: standard error of the mean; n=3). Note that expression of some genes is not significantly changed in response to S100A1 (e.g. fibronectin 1). **C**, Representative Ca^{2+} transients of adult rat cardiomyocytes incubated with PBS (control) or S100A1 1μM for 12hrs. Measurements were performed under basal conditions and stimulation with 1nM isoproterenol. Incubation with S100A1 had no influence on diastolic Ca^{2+} levels or Ca^{2+} transient amplitude. **D**, Pre-incubation of adult cardiomyocytes with S100A1 had no effect on susceptibility to caffeine (caff) or Camptothecin (CAM) induced apoptosis.
