# Supplementary material for: S100A1 is released from ischemic cardiomyocytes and signals myocardial damage via Toll-like receptor 4
Source: EMBO Mol Med. 2014 May 15;6(6):778–94. doi: 10.15252/emmm.201303498 (PMC4203355; doi:10.15252/emmm.201303498)
Supplement: Supplementary file 8 — Supplementary Figure S8 [file emmm0006-0778-sd8.pdf]

**Supplemental Figure VIII, Rohde et al.**

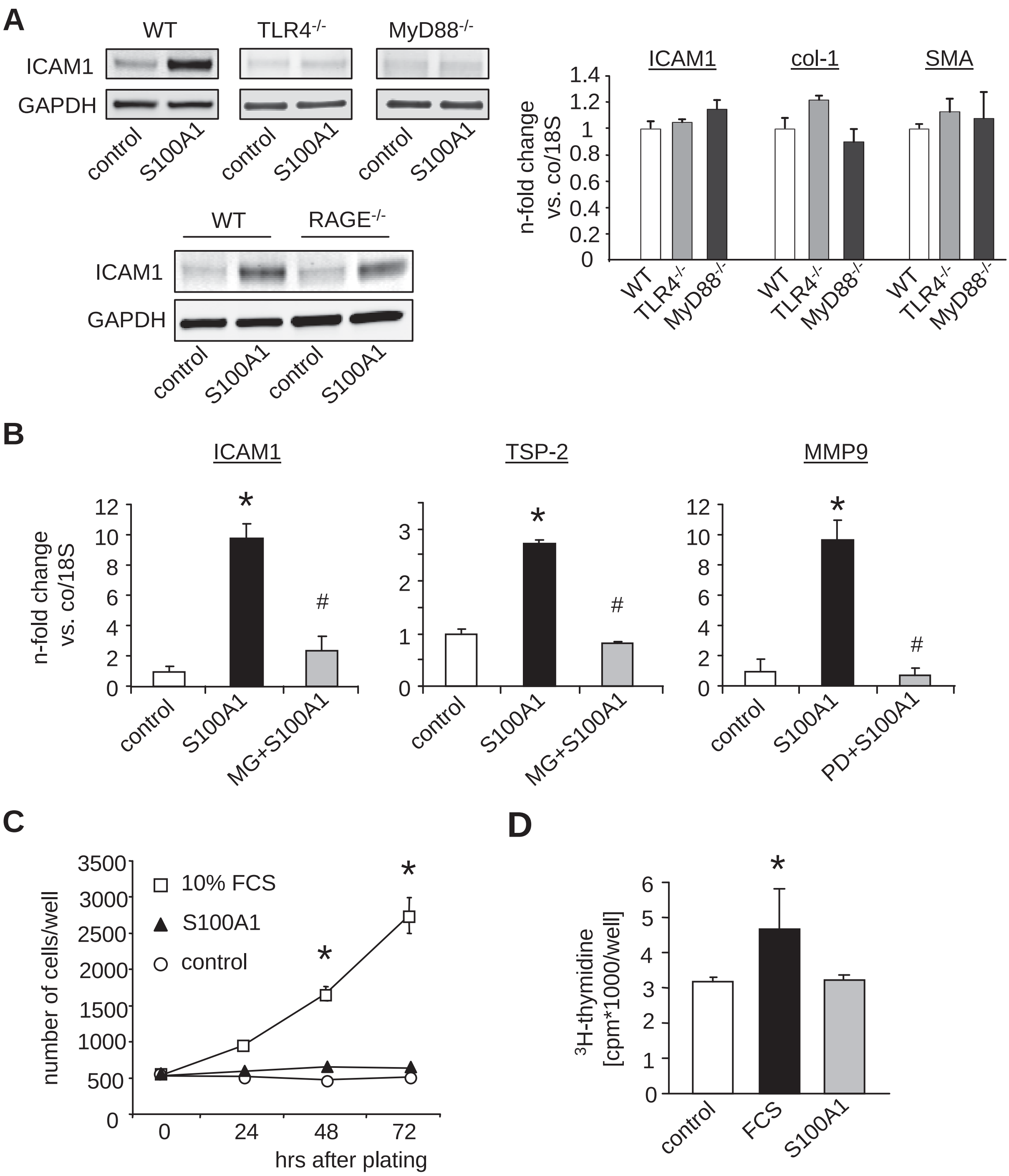


**Supplemental Figure VIII. Analysis of S100A1-mediated gene expression modifications and S100A1 influence on proliferation of cardiac fibroblasts. A**, S100A1-related upregulation of ICAM1 is blunted in TLR4 and MyD88 knock out fibroblasts (TLR4$^{-/-}$, MyD88$^{-/-}$) but not in RAGE knock out fibroblasts (RAGE$^{-/-}$) (left panel: representative Western blots, right panel: RT-PCR results, n=3). **B**, S100A1-mediated ICAM1 and thrombospondin 2 (TSP-2) upreguation is abrogated by pre-incubation with NFkappaB inhibitor MG-132 (MG). Pre-treatment of cardiac fibroblasts with the ERK1/2 inhibitor PD98059 (PD) results in suspension of MMP9 upregulation in response to S100A1 (n=5; */#P-values vs co/S100A1: 0.002/0.01 for ICAM1, 0.02/0.01 for TSP-2, 0.01/0.003 for MMP9). **C-D**, Incubation of cardiac fibroblasts with S100A1 had no effect on proliferation measured as increase in cell number (**C**) or amount of incorporated $^{3}$H-thymidine (**D**). Stimulation with FCS served as positive control for increased fibroblast proliferation (n=3; **C**: *P-value vs co 0.001 for 48 and 72hrs; **D**: *P=0.03 vs co).
