# Supplementary material for: S100A1 is released from ischemic cardiomyocytes and signals myocardial damage via Toll-like receptor 4
Source: EMBO Mol Med. 2014 May 15;6(6):778–94. doi: 10.15252/emmm.201303498 (PMC4203355; doi:10.15252/emmm.201303498)
Supplement: Supplementary file 9 — Supplementary Figure S9 [file emmm0006-0778-sd9.pdf]

Supplemental Figure IX, Rohde et al.

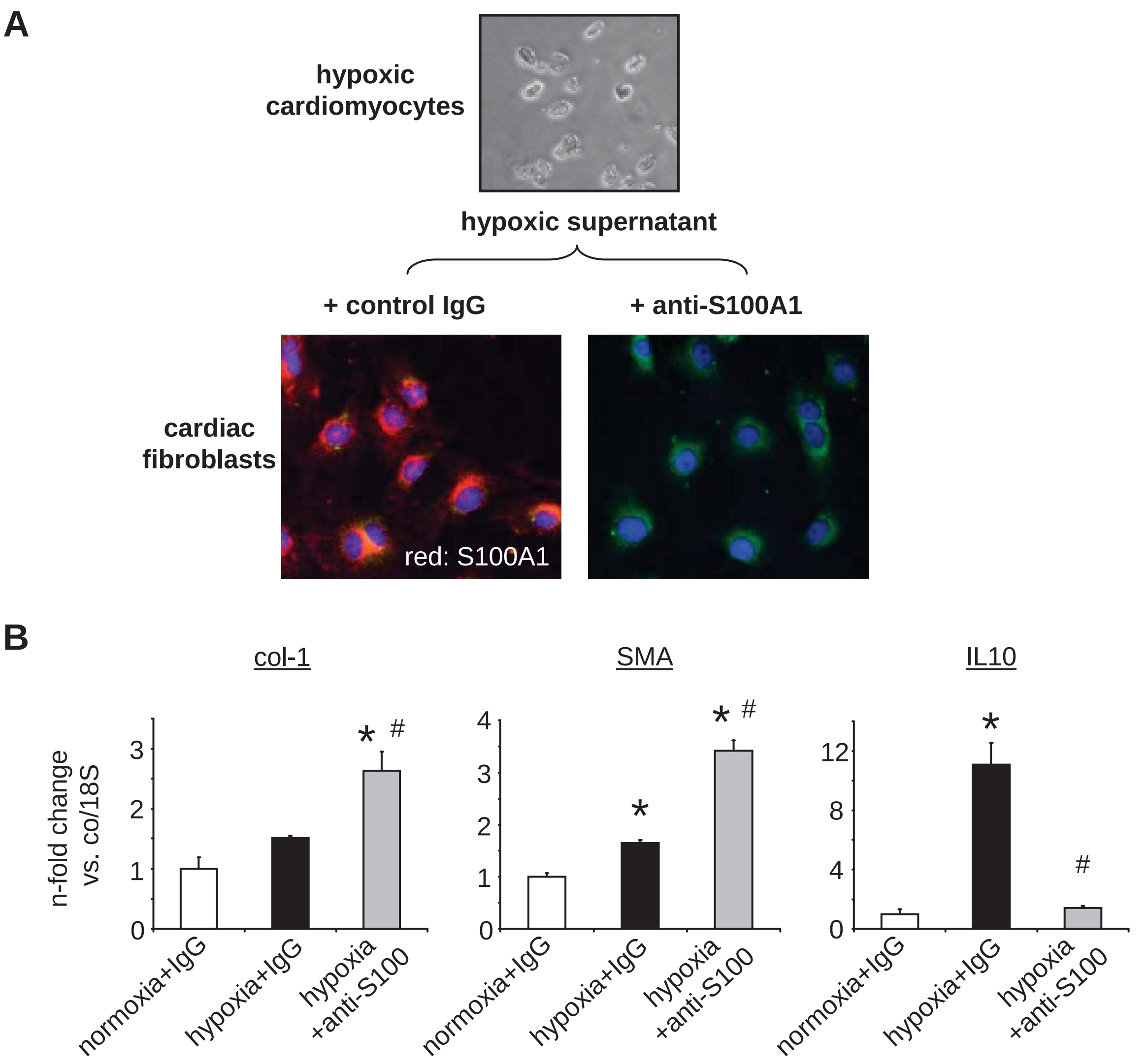

Supplemental Figure IX. Neutralization of S100A1 in necrotic cardiomyocyte supernatant by anti-S100A1 antibody. A, Supernatant taken from hypoxic cardiomyocytes (brightfield image) was treated with unspecific control IgG or anti-S100A1 antibody prior to incubation with cardiac fibroblasts (immunofluorescence images). Pre-treatment of the supernatant with anti-S100A1 abrogated internalization of S100A1 in cardiac fibroblasts (right immunofluorescence image) (S100A1: red, endoplasmic reticulum: green, DAPI/nuclei: blue). **B**, Cardiac fibroblasts were exposed to supernatants from normoxic or hypoxic cardiomyocytes (normoxia, hypoxia, respectively). Normoxic cardiomyocyte supernatant was treated with unspecific control IgG. Hypoxic cardiomyocyte supernatant was either treated with unspecific control IgG or anti-S100A1 antibody. Cardiac fibroblasts incubated with S100A1-depleted supernatant of hypoxic cardiomyocytes (hypoxia+anti-S100) responded with exaggerated pro-fibrotic marker expression (col-1: collagen type 1; SMA: smooth muscle actin) together with abrogated interleukin 10 (IL10) upregulation (n=5; *P-values vs normoxia: 0.02 for col-1, 0.04/IgG and 0.01/anti-S100 for SMA, 0.01 for IL10, #P-values vs hypoxia with control IgG: 0.03 for col-1, 0.02 for SMA, 0.01 for IL10).
