# Supplementary material for: S100A1 is released from ischemic cardiomyocytes and signals myocardial damage via Toll-like receptor 4
Source: EMBO Mol Med. 2014 May 15;6(6):778–94. doi: 10.15252/emmm.201303498 (PMC4203355; doi:10.15252/emmm.201303498)
Supplement: Supplementary file 10 — Supplementary Figure S10 [file emmm0006-0778-sd10.pdf]

Supplemental Figure X, Rohde et al.

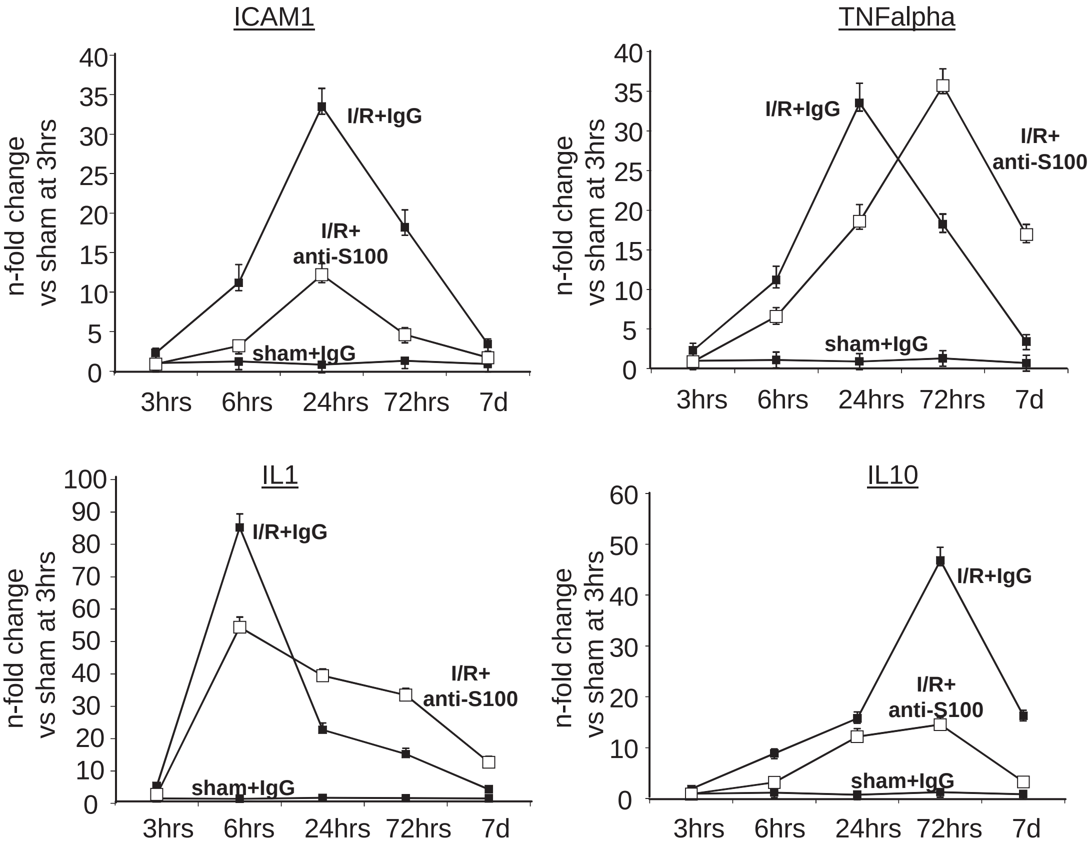

Supplemental Figure X. Effect of S100A1 neutralization on myocardial expression levels of inflammtory genes at different time points post ischemic injury *in vivo*. Mice were pre-treated with control-IgG or anti-S100A1 and ischemia/reperfusion injury (I/R) was conducted as previously described (small black squares: sham operation with injection of control-IgG, big black squares: I/R with injection of control-IgG, big white squares: I/R with injection of anti-S100A1). At the indicated time points, mice were sacrificed and left-ventricular myocardial tissue analysed for gene expression using RT-PCR (n=4 animals in each group, data presented as mean±SEM). Hightened TNFalpha and IL1 expression in the anti-S100A1-treated group might indicate a prolonged and potentially unfavourable myocardial inflammation in response to ischemic injury.
