# Supplementary material for: S100A1 is released from ischemic cardiomyocytes and signals myocardial damage via Toll-like receptor 4
Source: EMBO Mol Med. 2014 May 15;6(6):778–94. doi: 10.15252/emmm.201303498 (PMC4203355; doi:10.15252/emmm.201303498)
Supplement: Supplementary file 11 — Supplementary Methods [file emmm0006-0778-sd11.pdf]

<u>Supplementary Information</u>

Rohde D et al., S100A1 is released from ischemic cardiomyocytes and signals myocardial damage via Toll-like receptor 4

SUPPLEMENTARY METHODS

Patients

Patients presenting with acute chest pain to the internal medicine emergency department were screened. All consecutive patients that were classified as ST-segment elevation myocardial infarction (according to (Thygesen et al, 2012)) qualified for enrolment. All medical decisions including the need and timing of coronary angiography, coronary intervention or further diagnostic work-up were left at the discretion of the attending cardiologist. However, all patients underwent underwent coronary angiography and received a percutaneous coronary intervention (PCI). Blood samples were taken 8-12hrs after clinical onset of symptoms regardless of the time point of percutaneous coronary intervention or the exact coronary pathology. For the control group, patients presenting with non-cardiac symptoms (e.g. respiratory infection without serious dyspnea) were enrolled. Acute myocardial infarction was excluded in this group (according to (Thygesen et al, 2012)). Patients with known but stable coronary artery disease or other cardiac pathology were not excluded. Blood samples were taken in the course of in-hospital treatment. Patients with Troponin T elevation without myocardial infarction were not excluded. For further characteristics of STEMI patients and controls see Supplemental Figure IA. The study was performed according to the principles of the Declaration of Helsinki and approved by the local ethics committee. Written informed consent was obtained from all participating patients.

Laboratory measurements

Cardiac troponin was measured on COBAS E411 using the novel high-sensitive Troponin T assay (Roche Diagnostics Ltd., Rotkreuz, Switzerland) (Giannitsis et al, 2009). Limit of blank (3ng/L) and limit of detection (5ng/L) were determined in accordance with CLSI guideline EP17-A. The inter-assay coefficient of variation (CV) was 8% at 10ng/L and 2.5% at 100 ng/L. The intra-assay CV was 5% at 10ng/L and 1% at 100ng/L. Normal reference values were established from a multicentre reference study and the 99th percentile value was determined at 14ng/L (Saenger et al, 2011). Creatin kinase (CK) and lactate dehydrogenase (LDH) values were determined as part of the diagnostic routines established in the central laboratory unit of Heidelberg University Hospital.

Assessment of S100A1 serum levels

Detection of S100A1 was performed by custom-made enzyme-linked immunosorbent assay (ELISA). Serum samples were centrifuged at 5.000rpm for 10min at room temperature. The supernatant was collected and stored at -20°C. For ELISA measurement, a microtiter plate (Maxisorb, Nunc) was coated with the capture antibody (anti-S100oα rabbit polyclonal, ab11428, abcam). Therefore 100µL of a capture antibody solution (2µg/mL in coating solution, #80050, Alpha Diagnostic International) were loaded into the wells and incubated at

4°C over night. Next day, each well was washed 3 times with wash solution (#80080, Alpha Diagnostic International). Thereafter, 300µL of blocking solution (#80060, Alpha Diagnostic International) were added. After incubation, 100µL of the serum samples (diluted 2:1 in sample diluent, HEPES 23.8g/L, BSA 10g/L, NaCl 5.84g/L, Tween-20 0.1% in ddH_2O) were added to the corresponding wells in dublicates and incubated at 4°C over night. A standard curve was included (2.8ng/mL, 5.6ng/mL, 28ng/mL, 56ng/mL, 140ng/mL of recombinant human S100A1). Next day, wells were washed and 100µL of the detection antibody (human S100A1 affinity purified polyclonal sheep IgG, R&D Systems) diluted 1:5000 in PBS (NaCl 8g/L, KCl 0.2g/L, Na_2HPO_4 1.42g/L, KH_2PO_4 0.245g/L, Tween-20 5% in ddH_2O) were added to the wells. After 3hrs of incubation at room temperature and consecutive washing, 100µL of a horseradish peroxidase conjugated revealing antibody (donkey anti-sheep IgG-HRP, sc-2473, Santa Cruz Biotechnology) diluted 1:1000 in PBS were added and the plate was incubated for 2hrs at room temperature. After wahsing, 100µL of TMB (3,3',5,5'-Tetramethylbenzidin)-substrate (#80091, Alpha Diagnostic International) were pipetted into the wells. After 20min of incubation, 50µL of stop solution (#80100, Alpha Diagnostic International) were added. The optical density of each well was measured with a multiplate reader (Multiskan Spectrum, Thermo Fisher Scientific) at 450nm and corrected at 570nm.

Mice

Male C57B/6 mice (WT) were purchased from Jackson Laboratory (Bar Harbor, ME). Receptor for advanced glycation endproducts knock out mice ($RAGE^{-/-}$) and corresponding WT controls were provided by Dr Bierhaus. Toll-like receptor 4 knock out mice ($TLR4^{-/-}$) and MyD88 knock out mice ($MyD88^{-/-}$) together with corresponding controls were provided by Dr Linke and Dr Kubatzky, respectively. All experiments were performed according to protocols approved by the *Institutional Animal Care and Use Committee* at Thomas Jefferson University and complied with the *Guide for the Care and Use of Laboratory Animals*.

Experimental myocardial infarction

Both ligation of the left anterior descending coronary artery (LAD) and ischemia/reperfusion procedure (I/R) were performed as previously described (Gao et al, 2010; Wang et al, 2009). In brief, mice were anesthetized and the heart was manually exposed through a small chest incision and a slipknot was made around the LAD 2-3mm from its origin with a 6.0 silk suture. Sham-operated animals were subjected to the same surgical procedures except that the suture was passed under the LAD but was not tied. In the I/R group, the slipknot was released and the myocardium was subjected to reperfusion after 30min of ischemia. For the analysis of S100A1 serum concentration, serial blood samples were taken from the left carotid artery of anesthetized animals.

Intramyocardial S100A1 injection

Mice were anesthetized and the heart was manually exposed as described above. 3 injections with 2-3µL each containing 10µM S100A1 were performed into the left ventricular apex. Control animals received the same injections with saline solution free from S100A1. Injection fluids were supplemented with Evan's Blue dye for visualization.

Antibody injection

Anesthetized mice were pre-treated with either a single i.p. injection of 200µg anti-S100A1 antibody (SA5632, custom-made and affinity-purified, Eurogentec, Cologne, Germany) or rabbit IgG (200µg) 6hrs prior to ischemia/reperfusion (I/R). Sham animals with rabbit IgG injection served as controls. 3 days after I/R mice were sacrificed and myocardial gene expression was assessed.

Echocardiography

In vivo left ventricular function was determined by echocardiography as described previously (Gao et al, 2010; Wang et al, 2009). Mice were anesthetized with 1.5% isoflurane and two-dimensional echocardiographic views of the mid-ventricular short axis were obtained at the level of the papillary muscle tips below the mitral valve (Vevo 770, VisualSonic, Toronto, Canada). LV ejection fraction (LVEF) was calculated as previously reported.

Determination of myocardial infarct size

Myocardial infarct size was determined by Evans blue-TTC double staining as described previously (Gao et al, 2010; Wang et al, 2009). Briefly, following 48hrs of reperfusion, the ligature around the coronary artery was re-tied and 0.2ml 2% Evans blue dye was injected into the left ventricular cavity. The heart was then quickly excised, frozen with dry ice and sliced into five 1.2mm-thick slices that were perpendicular to the long axis of the heart. The unstained portion of myocardium (area at risk, AAR) was separated from the Evans blue-stained portion of the myocardium (area not at risk, ANAR). The slides were then incubated in 1% TTC for 15min and then digitally photographed. The Evans blue–stained area (ANAR), TTC-stained area, and TTC-negative staining area (infarcted myocardium) were measured using the computer-based image analyzer SigmaScan Pro 5.0 (SPSS Science). Myocardial infarct size was expressed as a percentage of the infarcted area over AAR.

Immunofluorescence of heart sections

Immunofluorescence of tissue sections was performed as previously reported (Most et al, 2012). In brief, mouse hearts were excised 48hrs after myocardial infarction, flash frozen and sectioned (10µm). Sections were fixed in MeOH:Acetone (1:1), blocked and incubated with anti-S100A1 (SA5632, Eurogentec) and anti-DDR2 (sc-7555, Santa Cruz Biotechnology) antibodies. Following incubation with corresponding secondary antibodies, all specimen were imaged at 40x using a Sensicam highresolution camera and Streampix image software (Norpix) with the same illumination and acquisition conditions. Conversion to binary images was done using ImageJ.

Isolation of mouse heart cardiomyocyte and non-cardiomyocyte fraction

Cells were enzymatically isolated as described below following a previously published protocol (Volkers et al, 2010). Briefly, mice were anesthetized using isoflurane, euthanized by excision of the heart and subsequently the ascending aorta was fixed to a perfusion cannula using a 6F silk ligature. The hearts were then perfused retrogradely with tyrodes solution (HEPES 25mM, NaCl 130mM, NaH2PO4 10mM, $MgCl_2$ 2.5mM, KCl 27mM) supplemented with 0.2% glucose and 1mM 2, 3-butandione monoxime (BDM) (Sigma-Aldrich) at a flow-rate of 3mL/min for approximately 30sec before switching to tyrodes solution with BDM containing collagenase type II (Worthington Inc., Lakewood, New Jersey). When digested, hearts were cut into small pieces, transferred to a tube containing tyrodes solution supplemented with BDM, 5% fetal calf serum (FCS), and processed to a suspension using a transfer pipette. The suspension was then filtered through a mesh before cardiac myocytes were allowed to completely pellet and further designated as cardiomyocyte fraction. The supernatant was saved, pelleted at 10.000g for 5min and designated as non-cardiomyocyte fraction. Both fractions were treated with lysis buffer (PBS, pH 7.4, SDS 1%, and 1mM EGTA/EDTA) containing a mixture of 1% (v/v) phosphatase inhibitors (Phosphatase Inhibitor Cocktail I and III, Sigma-Aldrich) and protease inhibitor (1 tablet/10mL) (Mini Complete EDTAfree protease inhibitor, Roche Applied Science) and subjected to Western blot analysis.

Cell isolation and culture

Cardiomyocytes were isolated from adult rats by a standard enzymatic digestion procedure and cultivated as described (Most et al, 2004). Adult rat cardiac fibroblasts (CFs) were obtained from the supernatant of cardiomyocytes and immunofluorescent staining for the fibroblast-specific discoidin domain receptor 2 (DDR2) yielded more than 99% CFs (Goldsmith et al, 2004). Cells were used between passage 2 and 3. For isolation of murine ear fibroblasts (MEFs) mice were sacrificed and ears were removed. Using a scalpel, epidermis and hair were gently scratched off the ears. Thereafter tissue was cut into small pieces and incubated with collagenase typ II (2mg/mL, dissolved in serum-free DMEM) for 45min at 37°C. Tissue was collected by centrifugation (1200rpm, 5min), resuspended in trypsin solution (0.05% in PBS, PAA Laboratories) and incubated for 45min at 37°C. Tissue was collected again by centrifugation, resuspended in DMEM containing 20% FCS and dissociated by repeated aspiration using a needle (18-gauge) and syringe. Finally tissue suspension was filtered through a 10µm nylon cell strainer and cell suspension was seeded in a cell culture dish coated with gelatine. CFs and MEFs were grown in DMEM with high glucose (PAA Laboratories) in the presence of 10% fetal calf serum (PAA Laboratories), 1% L-Glutamine (PAA Laboratories) and 1% penicilline/streptomycine (PAA Laboratories). Cells were pre-incubated with the tested inhibitors for 30min and then further incubated with S100A1 or other reagents.

Expression and purification of recombinant human S100A1 protein

Human recombinant S100A1 protein was produced in Escherichia coli as described previously (Most et al, 2003). The purified protein contained an endotoxic activity of about 25EU/mg as determined by the Limulus amoebocyte lysate kit (QCL-1000, BioWhittaker, Walkersville, MD). Subsequent purification applying EndoTrap removal columns (Hyglos, Bernried, Germany) yielded a reduction to approx. 1.1EU/mg S100A1 protein. *In vitro* S100A1 protein concentrations used in this study ranged from 0.01-10mM, thus corresponding to 0.0011-0.11EU/mL. Coupling of S100A1 protein to tetramethyl-rhodamin (TAMRA) was carried out by Eurogentec (Cologne, Germany).

Western blot

Western blotting was performed as previously reported (Most et al, 2012) to assess cardiac protein levels of S100A1 (SA5632, Eurogentec; 1:10000), GAPDH (MAB374, Millipore, 1:20000), p38 (#9212, Cell Signaling Technology, 1:1000), phospho-p38 (#9211, Cell Signaling Technology, 1:1000), ERK1/2 (#9102, Cell Signaling Technology, 1:5000), phospho-ERK1/2 (#9106, Cell Signaling Technology, 1:5000), JNK (#9252, Cell Signaling Technology, 1:1000), phospho-JNK (#9255, Cell Signaling Technology, 1:1000), actin beta (A5441, Sigma-Aldrich, 1:10000), p65 (#3034, Cell Signaling Technology, 1:1000), phospho-p65 (#3036, Cell Signaling Technology, 1:1000), ICAM1 (sc-1511, Santa Cruz Biotechnology, 1:1000), thrombospondin 2 (sc-12313, Santa Cruz Biotechnology, 1:1000), Akt (#9272, Cell Signaling Technology, 1:1000), phospho-Akt (#4051, Cell Signaling Technology, 1:1000), STAT3 (#9139, Cell Signaling Technology, 1:1000), phospho-STAT3 (#9131, Cell Signaling Technology, 1:1000) and HMGB1 (ab18256, abcam, 1:1000). Briefly, cultered cells were rinsed in PBS and scraped off the dish in lysis buffer (PBS, pH 7.4, SDS 1%, and 1mM EGTA/EDTA) containing a mixture of 1% (v/v) phosphatase inhibitors and protease inhibitor. Protein content was assessed employing the Bio-Rad DC protein assay (Bio-Rad Laboratories). After separation of protein samples (50µg per lane) by 4-20% SDS-PAGE (Invitrogen), proteins were transferred to a PVDF membrane and probed with an appropriate primary antibody at 4°C overnight. After staining with a corresponding pair of Alexa Fluor 680- (Molecular Probes; 1:10000) and IRDye 800-coupled (Rockland Inc.; 1:10000) secondary antibody, respectively, for 2hrs at room temperature proteins were

visualized with an Odyssey infrared imager (LI-COR Biosciences), and semiquantitative densitometric analysis was performed applying Odyssey version 1.2 infrared imaging software. Signals were normalized to GAPDH or actin beta densitometric levels that were not different between groups. Comparability between gels was obtained with data from 2 samples that were run on every gel.

Electrophoretic mobility shift assay (EMSA)

EMSA was performed as decribed previously (Bierhaus et al, 1995; Rudofsky et al, 2007). Briefly, nuclear proteins were prepared by the method of Andrews et al.(Andrews & Faller, 1991) For the NFkappaB binding reaction, 10µg of nuclear protein were mixed with radio labeled NFkappaB oligonucleotides (5´-AGT TGA GGG GAC TTT CCC AGG C-3´). After incubation at room temperature, protein-DNA complexes were separated from unbound nucleotides by electrophoresis through native 5% polyacrylamide gels. After drying, gels were exposed to Amersham Hyperfilms and densitometric quantification was carried out by using LI-COR Odyssey Software. The specifity of the binding reaction was assessed by competition with a 160-fold molar excess of unlabeled oligonucleotides. For supershift analysis, 2.5µg of the respective NFkappaB antibody (anti-p50, -p65, -cRel, -RelB, -p52) were applied to the binding reaction (NFkappaB antibodies were obtained from Santa Cruz Biotechnology).

RNA isolation, reverse transcription and semiquantitative real-time polymerase chain reaction (RT-PCR)

Total RNA isolation from LV tissue samples was performed applying the TRIZOL method, according to the manufacturer's protocol (Invitrogen) as previously described (Most et al, 2012). Quality of RNA was assessed by running an aliquot on a denaturing 1% agarose gel. First strand cDNA synthesis from 1µg of total RNA was carried using iScript cDNA Synthesis Kit (Bio-Rad Laboratories). For RT-PCR, 5µL of diluted cDNA (1:100) were added to iQ SYBR Green Supermix (Bio-Rad Laboratories) and 100nM of gene-specific oligonucleotides. Subsequently, quantitative PCR was carried out on a MyiQ Single-Color Real-Time PCR detection system (Bio-Rad Laboratories). For SYBR Green PCR based expression profiling, the rat inflammatory cytokines & receptors (PARN-011) and extracellular matrix and adhesion molecules (PARN-013) RT-PCR arrays from SABiosciences were employed according to the manufacturer's protocol (for a complete gene list see www.sabiosciences.com). For selected genes, results were validated by alternative gene-specific oligonucleotide primers generated by the use of PRIMER3 software (col-1: fwd 5'-GAG CGG AGA GTA CTG GAT CGT-3' rev 5'-CTG ACC TGT CTC CAT GTT GCA-3', ICAM1: fwd 5'-CTG TCG GTG CTC AGG TAT CC-3' rev 5'-CCA ACT TCT CAG TCA CCT CC-3') or purchased from SABiosciences (CTGF PPR46426A, IL10 PPR06479A, MMP9 PPR44728C, SMA PPR59337A, TSP-2 PPR51039A, SDF1 PPR06722B). SYBR Green based semiquantitative PCR with gene-specific oligonucleotides was carried out in duplicate. After each run, saturation of each amplification cycle was controlled by the use of MyiQ software (version 1.0) and, subsequently, a melting curve was acquired by heating the product to 95°C, cooling to and maintaining at 55°C for 20 seconds, then slowly (0.5°C/s) heating to 95°C in order to determine the specificity of the PCR products, which were then confirmed by agarose gel electrophoresis.

Immunofluorescence of cells

In vitro immunofluorescence imaging was essentially performed as previously described (Most et al, 2012). Cells were seeded overnight on gelatine-coated glass coverslips, fixed with 4% paraformaldehyde and permeabilized using Triton X-100 (Sigma-Aldrich). After incubation with appropriate primary and secondary antibodies or labelled phalloidine

(Invitrogen), coverslips were mounted using Vectashield medium with DAPI (Vector Laboratories). Images were obtained with an Olympus IX81 microscope.

Life cell imaging (lysotracker, mitotracker)

Cardiac fibroblasts were seeded on cell culture dishes with glass bottom (Fluoro Dish, World Precision Instruments) over night. After cells were starved for 24hrs with DMEM containing 0,5% FCS, media was changed to DMEM containing 50nM lysotracker or mitotracker (Invitrogen). Immediately thereafter rhodamine-conjugated S100A1 was added (final well concentration 1μM). After incubation for 30min, cells were washed 3 times with PBS and covered with 1mL PBS. Images were obtained immediately using an Olympus IX81 microscope.

Proximity ligation assay (Duolink®)

The Duolink® assay was purchased from Olink Bioscience and performed according to the manufacturer's instructions as previously reported (Most et al, 2012). Adult rat cardiac fibroblasts were seeded on glass coverslips and fixed and permeabilized as described above. Cells were then incubated with the two antibodies of interest for 4hrs (S100A1: SA5632, custom-made and affinity-purified, Eurogentec, Cologne, Germany; TLR4: 5031A, Imgenex Corp.). After first antibody incubation, cells were washed with PBS and incubated for one hour with the corresponding PLA probes (plus and minus probes) diluted in blocking buffer 1:5. For detection, cells were washed again and a ligation-ligase mixture was added and incubated on each sample for 30min. After removal of the mixture and a washing step, an amplification-polymerase solution was added followed by an incubation period of 100min. After a final washing step, the samples were mounted (Vectashield with DAPI, Vector Laboratories) over night and subsequently imaged (Olympus IX81).

Gelatin zymography

Measurement of matrix metalloproteinase (MMP) activity was essentially performed as described previously (Siwik et al, 2000; Xie et al, 2004). In brief, unconditioned cell culture medium was mixed with sample buffer and directly loaded onto polyacrylamide gels polymerized with 0.1% gelatin as substrate. After electrophoresis under non-reducing conditions, gels were incubated with renaturating buffer for 30min followed by over night incubation in developing buffer. Subsequently, gels were stained in coomassie blue, scanned and densitometric quantification of unstained, digested regions representing MMP activity was assessed by using LI-COR Odyssey software. All reagents required for gelatin zymography were purchased from Invitrogen.

Interleukin 10-ELISA

Cell culture supernatant concentration of Interleukin 10 was measured by enzyme-linked immunosorbent assay according to the manufacturer's protocols (R1000, R&D Systems).

[^{3}H]proline incorporation assay

Measurement of total collagen synthesis was assessed by [^{3}H]proline incorporation as described in detail elsewhere (Brilla et al, 1994). Briefly, cardiac fibroblasts were grown to 70-80% confluence in 12-well plates in DMEM containing 20% FBS and serum-starved for 24hrs. Cultures were then exposed to control (vehicle) or stimulated conditions (0.1, 1 and 10μM S100A1 or 20% FBS) for 24-72hrs in the presence of 4μCi/mL [^{3}H]proline and 50μg/mL ascorbic acid. At the end of the respective incubation times, conditioned media proteins were precipitated with an equal volume of 12% TCA. The TCA-precipitated proteins were centrifuged at 1.000g for 10min. The resulting pellets were washed three times with 6%

TCA and then solubilized in 0.2N NaOH. Aliquots from each sample were counted in a Beckman scintillation counter. The remainder samples were adjusted to contain (in mM) 100 NaCl, 50 HEPES, and 3 $CaCl_2$ at pH 7.0. Collagenase type III (100U/mL) was then added to each sample, followed by incubation for 16hrs at room temperature. After collagenase digestion, the proteins were again precipitated as described above, washed three times with 6% TCA, solublized in 0.2N NaOH, and subjected to liquid scintillation counting. Collagenase-sensitive [^{3}H]proline incorporation was calculated as the difference between TCA precipitable counts before and after collagenase digestion, which reflects total collagen synthesis and expressed as % of control samples. Experiments were performed in triplicate and repeated with cells from 3 different preparations.

[^{3}H]thymidine incorporation assay

[^{3}H]thymidine incorporation was carried out as described recently (Most et al, 2012). Briefly, CFs were grown to 20-30% confluency in 6-well plates in DMEM containing 20% FBS and serum-starved for 24hrs. CFs were then exposed to control or stimulated conditions (0.1, 1 and 10μM S100A1 or 20% FBS) in DMEM containing 0.5% FBS for 24-48hrs. [^{3}H]thymidine (1μCi/mL, specific activity 20Ci/mmol) was added in the last 4hrs of incubation. For the assessment of [^{3}H]thymidine incorporation, media was removed at the end of incubation and cells were washed with 10% trichloroacetic acid (TCA) and digested with 0.5N NaOH. Radioactivity in the cell digest was counted in a Beckman scintillation counter. [^{3}H]thymidine incorporation is expressed as total counts per minute per well.

Proliferation assay

For the detection of proliferation rates, cardiac fibroblasts (passage 2) were trypsinized and automatically counted in suspension using a Cellometer Auto T4 (Nexcelom Bioscience, Lawrence, MA). 500.000 cells were then seeded into each well of 6-Well-Plates and S100A1 or FCS was added to the cell culture media in the appropriate groups. Following 24, 48 or 72hrs of incubation, cells were again trypsinized and automatically counted. Each experiment was carried out in quadruplicates, data from n=5 individual experiments are presented as mean±SEM.

Assessment of reactive oxygen species (ROS)

Cardiac fibroblasts were grown in cell culture dishes with glass bottom (World Precision Instruments). Cells were loaded with the ROS-sensitive fluorogenic probe 2',7'-dichlorodihydrofluorescin diacetate (DCFH-DA) (C6827, Molecular Probes) in DMEM containing 0.5% FBS for 30min at 37°C. Cells were then washed in PBS and cultured for further 30min to allow complete deesterification of the dye. DCFH-DA enters the cell and is deacetylated by cellular esterases to non-fluorescent DCFH, which is rapidly oxidized to highly fluorescent compound DCF by ROS. Fluorescence measurement was carried out using an inverse Olympus microscope (IX81) equipped with U-MWU and U-MNIB filter cubes and connected to a monochromator (Polychrome II, TILL Photonics, Gräfelfing, Germany). Excitation was performed at 485nm and emission was detected at 535nm. Data were analyzed with TILLVision software (TILL Photonics).

Intracellular Ca^{2+} transients

Measurement of intracellular Ca^{2+} transients followed a previously published protocol (Volkers et al, 2010). Adult rat cardiomyocytes were loaded with Fura2-AM (2μM for 20min at 37°C to allow complete desterification of the dye). Measurements were carried out using an inverse Olympus microscope (IX81) with a UV filter connected to a monochromator (Polychrome II, TILL Photonics). Cells were electrically stimulated with a biphasic pulse to contract (2Hz) and excited at 340/380nm. Epifluorescence emission was detected at 510nm,

digitized, and analyzed off-line with TILLVision software. Baseline data from 10 consecutive steady-state transients after 15min of electrical stimulation were averaged for analysis of transient amplitude. Per group, cells from 5 different animals (approximately 30 cells per animal) were measured and data were pooled for analysis.

SUPPLEMENTARY INFORMATION REFERENCES

Andrews NC, Faller DV (1991) A rapid micropreparation technique for extraction of DNA-binding proteins from limiting numbers of mammalian cells. *Nucleic Acids Res* **19**(9)**:** 2499

Bierhaus A, Zhang Y, Deng Y, Mackman N, Quehenberger P, Haase M, Luther T, Muller M, Bohrer H, Greten J, et al. (1995) Mechanism of the tumor necrosis factor alpha-mediated induction of endothelial tissue factor. *J Biol Chem* **270**(44)**:** 26419-26432

Brilla CG, Zhou G, Matsubara L, Weber KT (1994) Collagen metabolism in cultured adult rat cardiac fibroblasts: response to angiotensin II and aldosterone. *J Mol Cell Cardiol* **26**(7)**:** 809-820

Gao E, Lei YH, Shang X, Huang ZM, Zuo L, Boucher M, Fan Q, Chuprun JK, Ma XL, Koch WJ (2010) A novel and efficient model of coronary artery ligation and myocardial infarction in the mouse. *Circ Res* **107**(12)**:** 1445-1453

Giannitsis E, Kurz K, Hallermayer K, Jarausch J, Jaffe AS, Katus HA (2009) Analytical validation of a high-sensitivity cardiac troponin T assay. *Clin Chem* **56**(2)**:** 254-261

Goldsmith EC, Hoffman A, Morales MO, Potts JD, Price RL, McFadden A, Rice M, Borg TK (2004) Organization of fibroblasts in the heart. *Dev Dyn* **230**(4)**:** 787-794

Most P, Boerries M, Eicher C, Schweda C, Ehlermann P, Pleger ST, Loeffler E, Koch WJ, Katus HA, Schoenenberger CA, Remppis A (2003) Extracellular S100A1 protein inhibits apoptosis in ventricular cardiomyocytes via activation of the extracellular signal-regulated protein kinase 1/2 (ERK1/2). *J Biol Chem* **278**(48)**:** 48404-48412

Most P, Lerchenmuller C, Rengo G, Mahlmann A, Ritterhoff J, Rohde D, Goodman C, Busch CJ, Laube F, Heissenberg J, Pleger ST, Weiss N, Katus HA, Koch WJ, Peppel K (2012) S100A1 deficiency impairs postischemic angiogenesis via compromised proangiogenic endothelial cell function and nitric oxide synthase regulation. *Circ Res* **112**(1)**:** 66-78

Most P, Pleger ST, Volkers M, Heidt B, Boerries M, Weichenhan D, Loffler E, Janssen PML, Eckhart AD, Martini J, Williams ML, Katus HA, Remppis A, Koch WJ (2004) Cardiac adenoviral S100A1 gene delivery rescues failing myocardium. *J Clin Invest* **114**(11)**:** 1550-1563

Rudofsky G, Jr., Reismann P, Grafe IA, Konrade I, Djuric Z, Tafel J, Buchbinder S, Zorn M, Humpert PM, Hamann A, Morcos M, Nawroth PP, Bierhaus A (2007) Improved vascular function upon pioglitazone treatment in type 2 diabetes is not associated with changes in mononuclear NF-kappaB binding activity. *Horm Metab Res* **39**(9)**:** 665-671

Saenger AK, Beyrau R, Braun S, Cooray R, Dolci A, Freidank H, Giannitsis E, Gustafson S, Handy B, Katus H, Melanson SE, Panteghini M, Venge P, Zorn M, Jarolim P, Bruton D, Jarausch J, Jaffe AS (2011) Multicenter analytical evaluation of a high-sensitivity troponin T assay. *Clin Chim Acta* **412**(9-10)**:** 748-754

Siwik DA, Chang DL, Colucci WS (2000) Interleukin-1beta and tumor necrosis factor-alpha decrease collagen synthesis and increase matrix metalloproteinase activity in cardiac fibroblasts in vitro. *Circ Res* **86**(12)**:** 1259-1265

Thygesen K, Alpert JS, Jaffe AS, Simoons ML, Chaitman BR, White HD, Katus HA, Lindahl B, Morrow DA, Clemmensen PM, Johanson P, Hod H, Underwood R, Bax JJ, Bonow RO, Pinto F, Gibbons RJ, Fox KA, Atar D, Newby LK, Galvani M, Hamm CW, Uretsky BF, Steg PG, Wijns W, Bassand JP, Menasche P, Ravkilde J, Ohman EM, Antman EM, Wallentin LC, Armstrong PW, Januzzi JL, Nieminen MS, Gheorghiade M, Filippatos G, Luepker RV, Fortmann SP, Rosamond WD, Levy D, Wood D, Smith SC, Hu D, Lopez-Sendon JL, Robertson RM, Weaver D, Tendera M, Bove AA, Parkhomenko AN, Vasilieva EJ, Mendis S (2012) Third universal definition of myocardial infarction. *Circulation* **126**(16)**:** 2020-2035

Volkers M, Weidenhammer C, Herzog N, Qiu G, Spaich K, von Wegner F, Peppel K, Muller OJ, Schinkel S, Rabinowitz JE, Hippe HJ, Brinks H, Katus HA, Koch WJ, Eckhart AD, Friedrich O, Most P (2010) The inotropic peptide betaARKct improves betaAR responsiveness in normal and failing cardiomyocytes through G(betagamma)-mediated L-type calcium current disinhibition. *Circ Res* **108**(1)**:** 27-39

Wang Y, Gao E, Tao L, Lau WB, Yuan Y, Goldstein BJ, Lopez BL, Christopher TA, Tian R, Koch W, Ma XL (2009) AMP-activated protein kinase deficiency enhances myocardial ischemia/reperfusion injury but has minimal effect on the antioxidant/antinitrative protection of adiponectin. *Circulation* **119**(6)**:** 835-844

Xie Z, Singh M, Singh K (2004) Differential regulation of matrix metalloproteinase-2 and -9 expression and activity in adult rat cardiac fibroblasts in response to interleukin-1beta. *J Biol Chem* **279**(38)**:** 39513-39519
