# Supplementary material for: S100A1 is released from ischemic cardiomyocytes and signals myocardial damage via Toll-like receptor 4
Source: EMBO Mol Med. 2014 May 15;6(6):778–94. doi: 10.15252/emmm.201303498 (PMC4203355; doi:10.15252/emmm.201303498)

Source Data - Figure 5 A Part 1

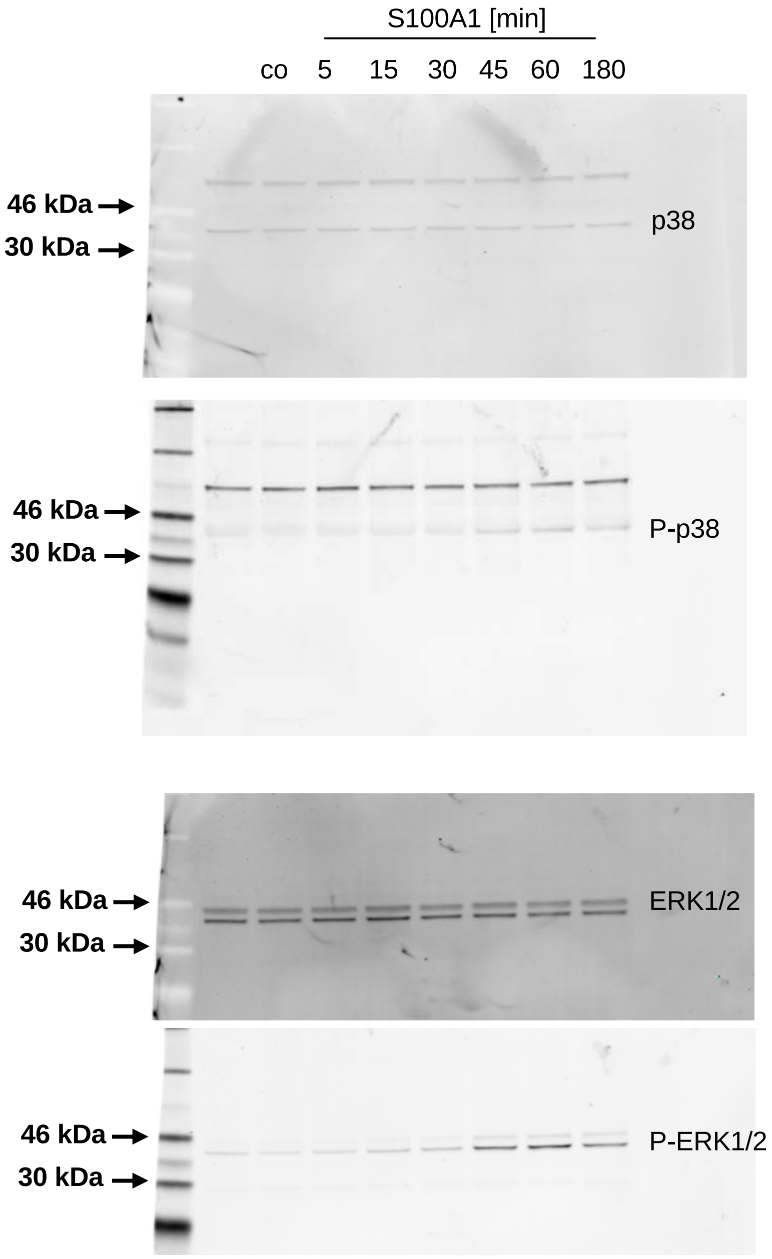

Source Data - Figure 5 A Part 2

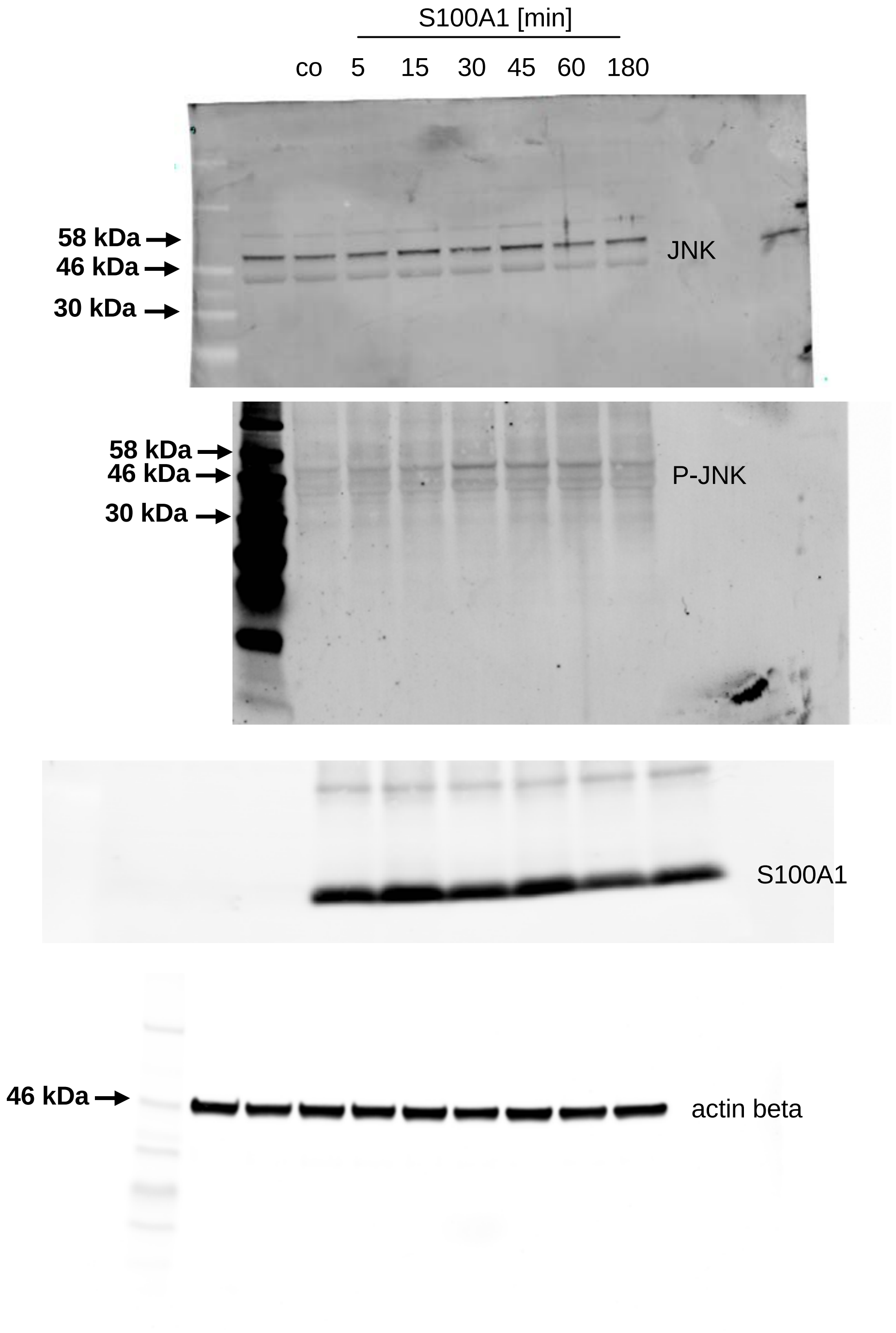

Source Data - Figure 5 B

S100A1 [min]

co 5 15 30 45 60 180 cons

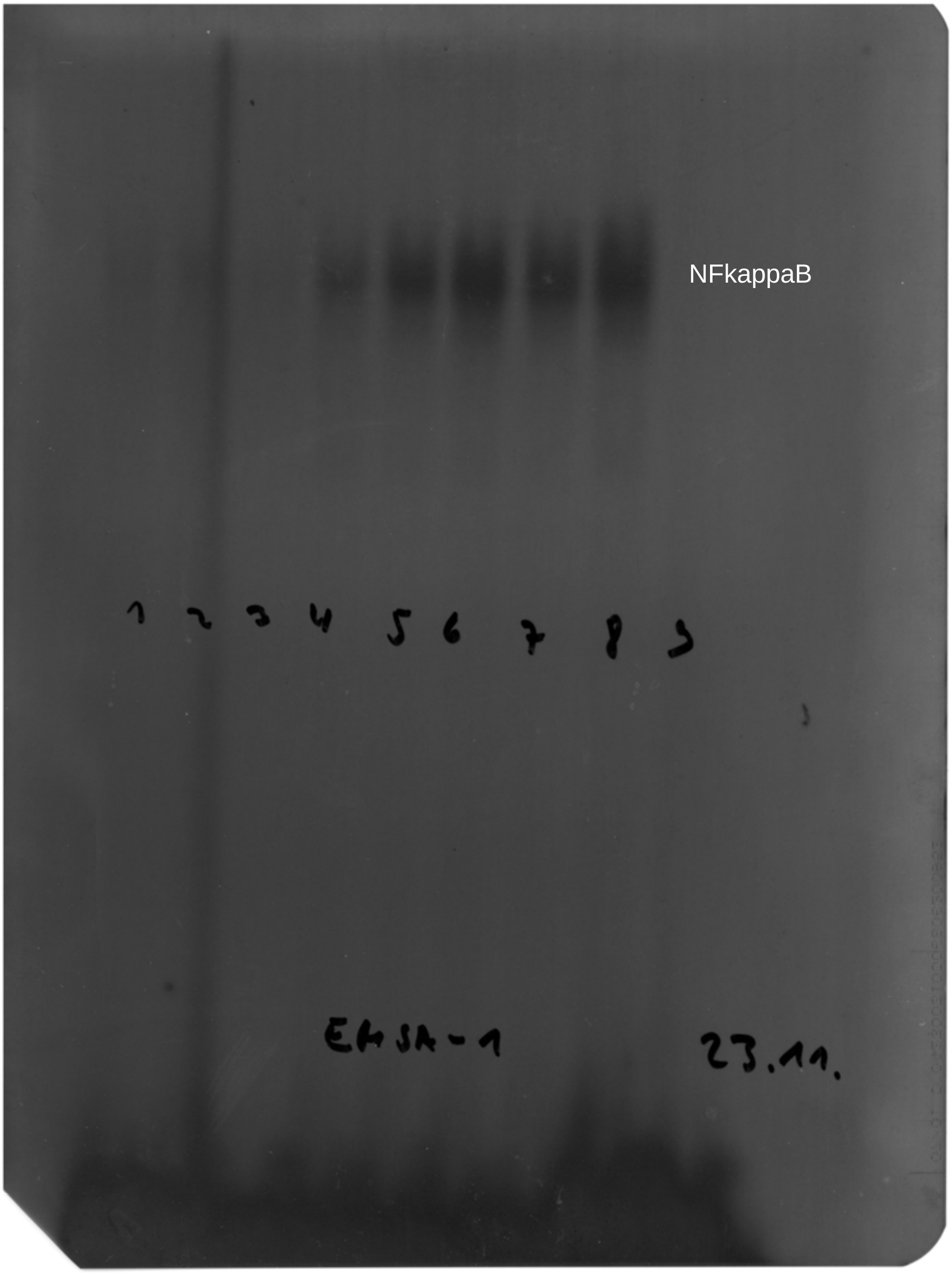

Source Data - Figure 5 C

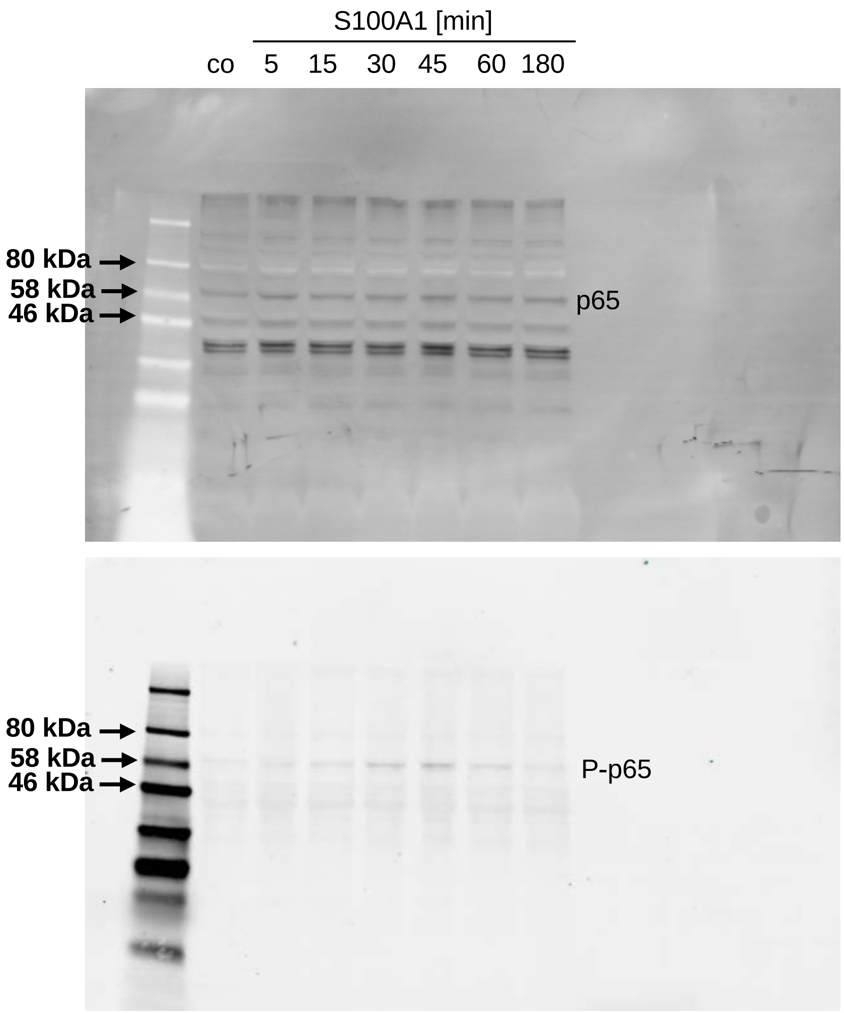

Source Data - Figure 5 E Part 1

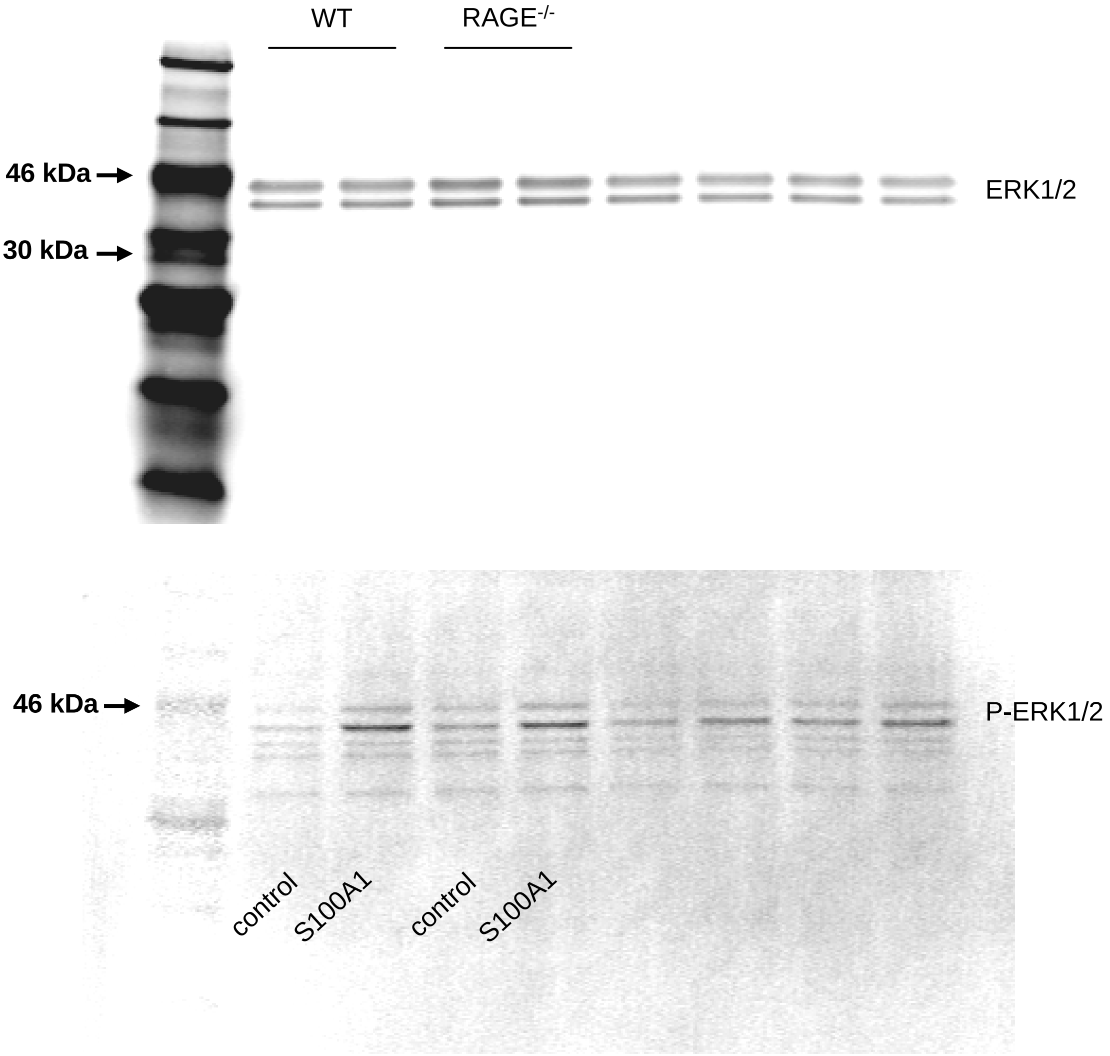

Source Data - Figure 5 E Part 2

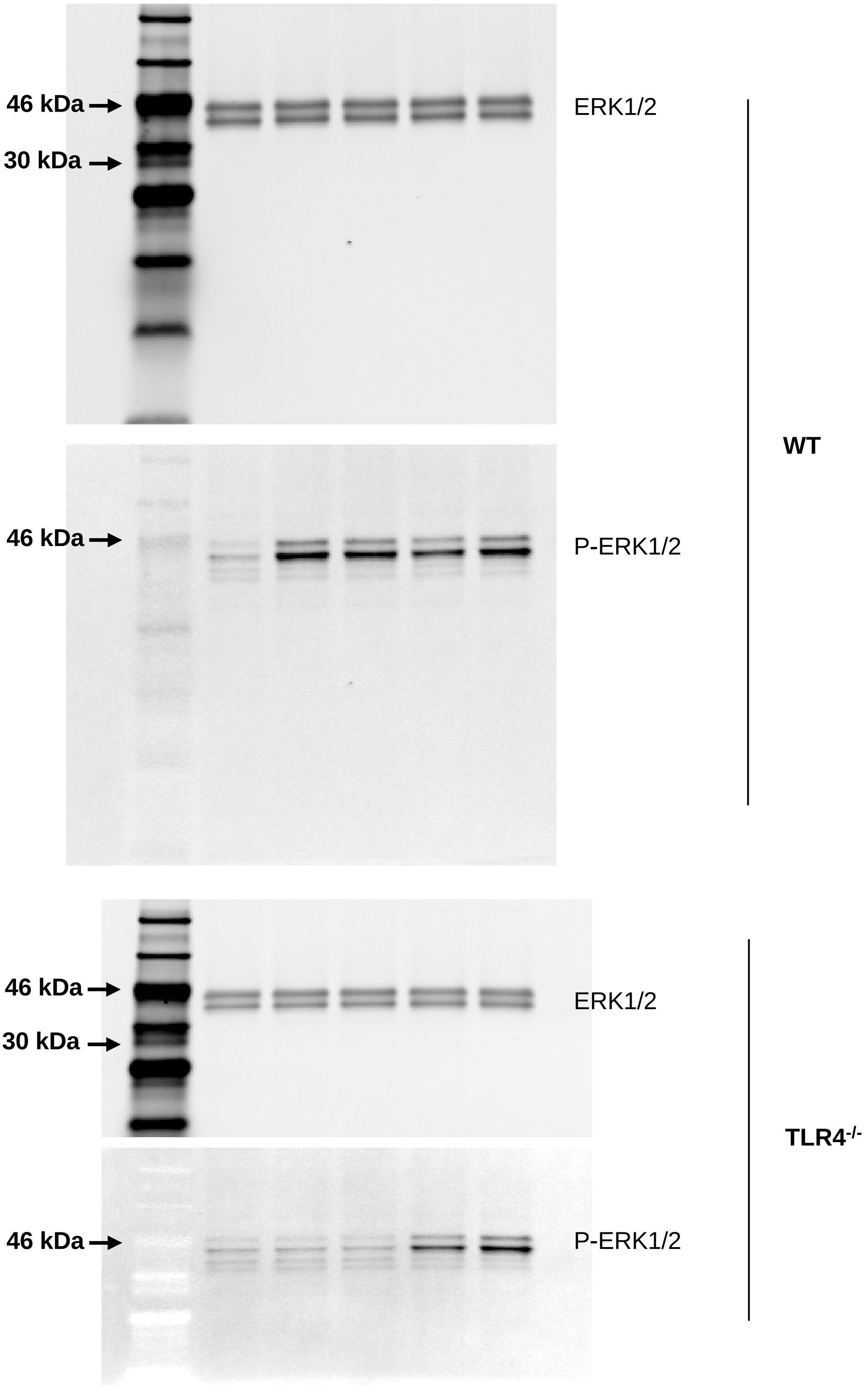

Source Data - Figure 5 E Part 3

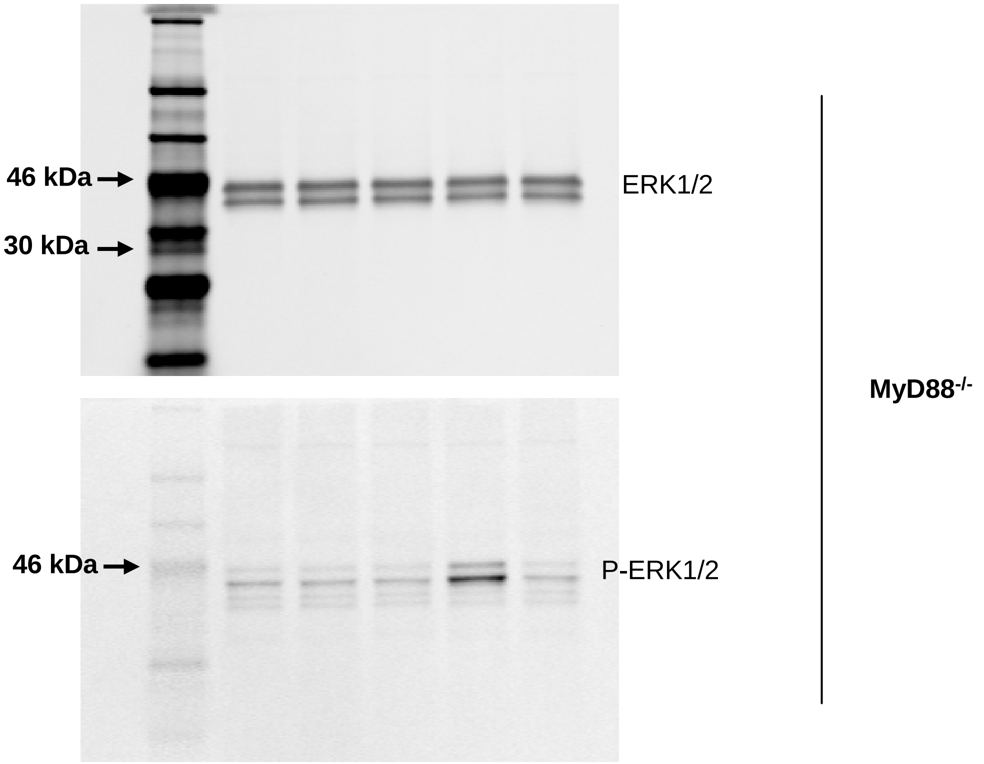

Source Data - Figure 5 F

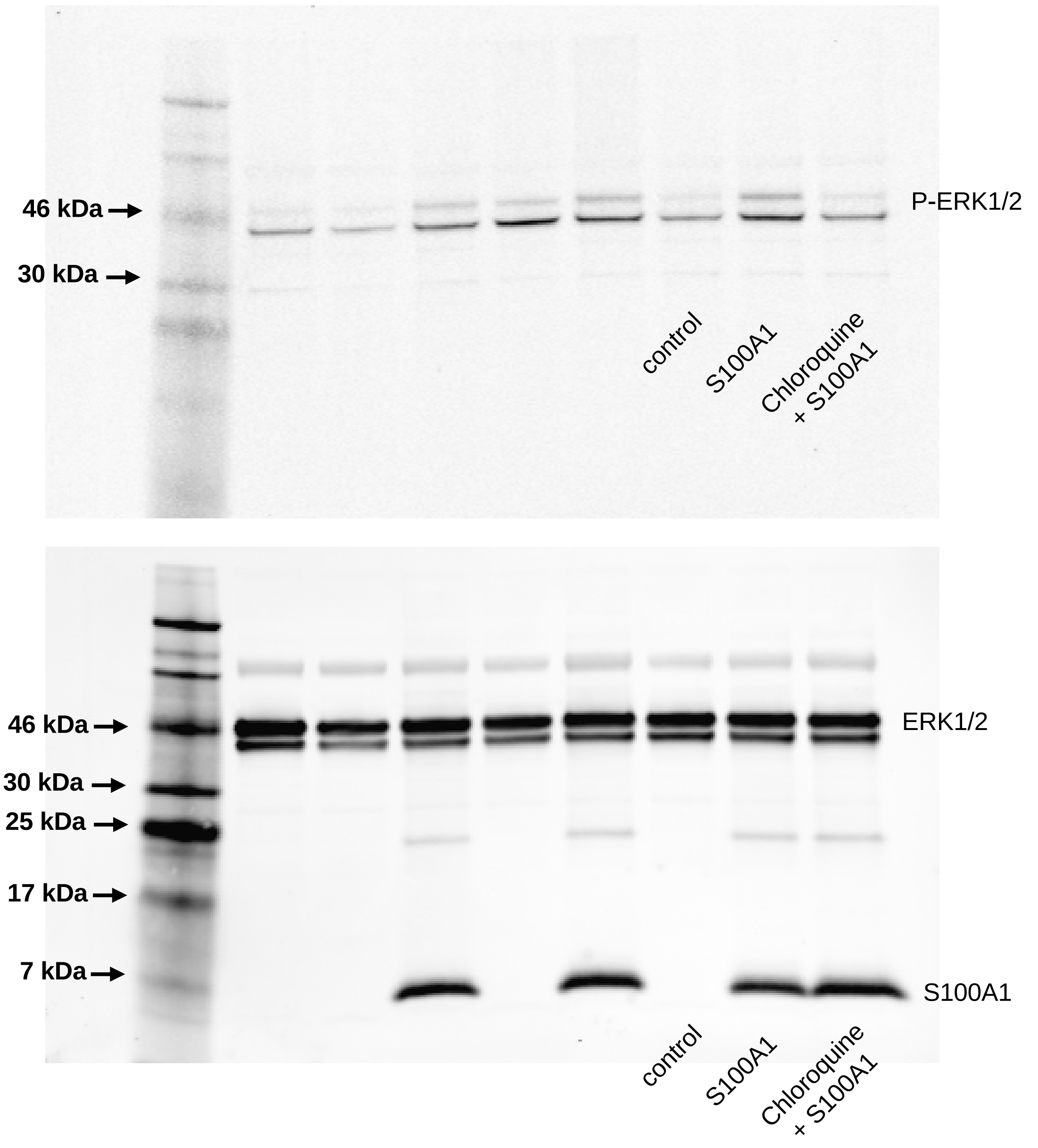

Supplement: Supplementary file 13 — Source data for Figure 5 A B C E F [file emmm0006-0778-sd13.pdf]
