# Supplementary figures and images for: S100A1 is released from ischemic cardiomyocytes and signals myocardial damage via Toll-like receptor 4
Source: EMBO Mol Med. 2014 May 15;6(6):778–94. doi: 10.15252/emmm.201303498 (PMC4203355; doi:10.15252/emmm.201303498)

Source Data - Figure 6 B

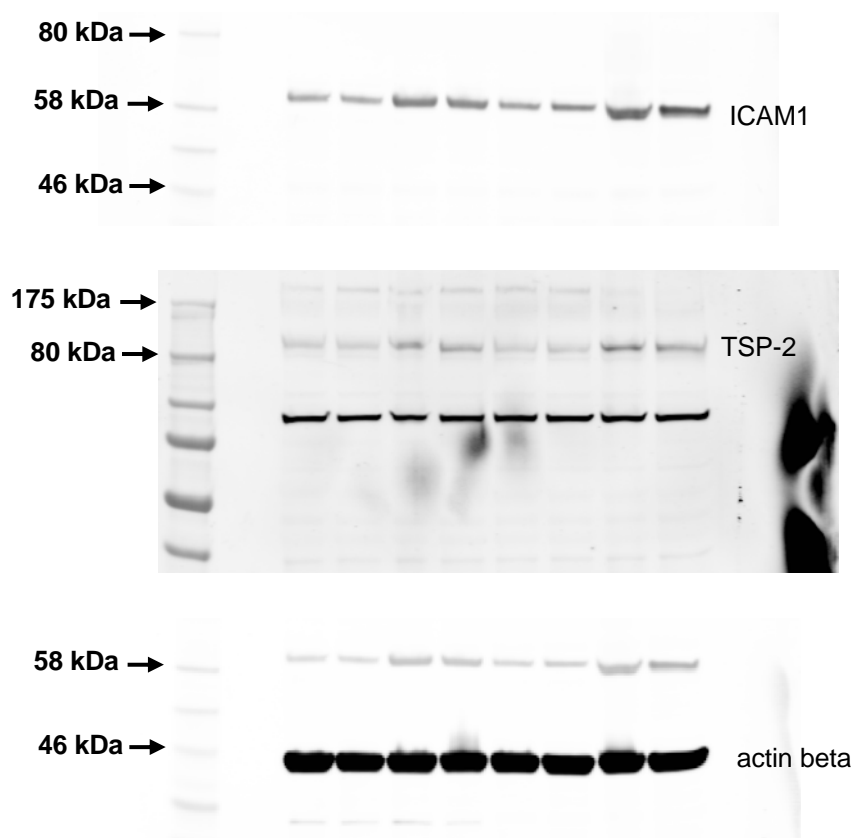

Source Data - Figure 6 F

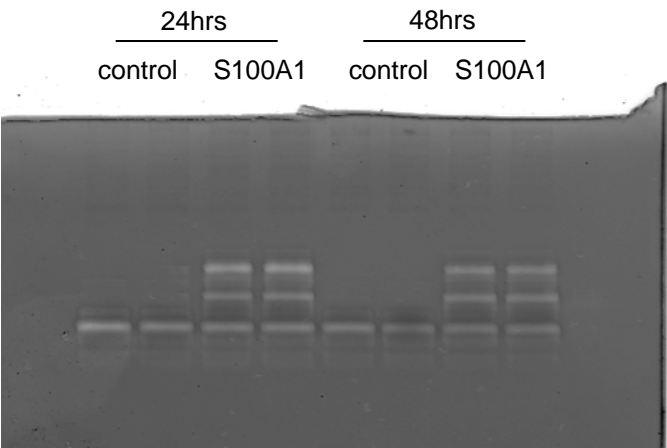

Supplement: Supplementary file 14 — Source data for Figure 6 B F [file emmm0006-0778-sd14.pdf]
